# Presence and role of viruses in anaerobic digestion of food waste under environmental variability

**DOI:** 10.1186/s40168-023-01585-z

**Published:** 2023-08-04

**Authors:** Lu Fan, Wei Peng, Haowen Duan, Fan Lü, Hua Zhang, Pinjing He

**Affiliations:** 1 Institute of Waste Treatment and Reclamation, Tongji University, Shanghai, 200092 China; 2 Shanghai Institute of Pollution Control and Ecological Security, Shanghai, 200092 China; 3 Shanghai Engineering Research Center of Multi-Source Solid Wastes Co-processing and Energy Utilization, Shanghai, 200092 China

**Keywords:** Anaerobic digestion, Food waste, Viruses, Ammonium, Long-chain fatty acids, Community, Auxiliary metabolic genes

## Abstract

**Background:**

The interaction among microorganisms in the anaerobic digestion of food waste (ADFW) reactors lead to the degradation of organics and the recycling of energy. Viruses are an important component of the microorganisms involved in ADFW, but are rarely investigated. Furthermore, little is known about how viruses affect methanogenesis.

**Results:**

Thousands of viral sequences were recovered from five full-scale ADFW reactors. Gene-sharing networks indicated that the ADFW samples contained substantial numbers of unexplored anaerobic-specific viruses. Moreover, the viral communities in five full-scale reactors exhibited both commonalities and heterogeneities. The lab-scale dynamic analysis of typical ADFW scenarios suggested that the viruses had similar kinetic characteristics to their prokaryotic hosts. By associating with putative hosts, a majority of the bacteria and archaea phyla were found to be infected by viruses. Viruses may influence prokaryotic ecological niches, and thus methanogenesis, by infecting key functional microorganisms, such as sulfate-reducing bacteria (SRB), syntrophic acetate-oxidizing bacteria (SAOB), and methanogens. Metabolic predictions for the viruses suggested that they may collaborate with hosts at key steps of sulfur and long-chain fatty acid (LCFA) metabolism and could be involved in typical methanogenesis pathways to participate in methane production.

**Conclusions:**

Our results expanded the diversity of viruses in ADFW systems and suggested two ways that viral manipulated ADFW biochemical processes.

Video Abstract

**Supplementary Information:**

The online version contains supplementary material available at 10.1186/s40168-023-01585-z.

## Background

Anaerobic digestion (AD) is an attractive energy production process that contributes to decarbonizing the economy and addresses climate change while successfully managing organic waste. The process can convert organic wastes, such as food waste (FW), agriculture waste, or activated sludge, into methane-rich biogas, known as important renewable energy [1].

The major steps in the AD process are hydrolysis, acidogenesis, acetogenesis, and methanogenesis. Microorganisms, both bacteria and archaea, are considered to be the main participants, with bacteria mainly catalyzing the first three steps and archaea converting small molecules into methane. Extensive research has been undertaken on the composition and behavior of prokaryotic communities in relation to biogas production efficiency and process stability in AD systems [2–4].

Recent developments in metagenomics have increased attention to viral ecology. Scientific researches on oceans [5], peatlands [6], soils [7], and wastewater treatment plants [8] have considerably expanded viral datasets and the cognition of global distribution characteristics. However, viruses have rarely been considered as an important component of AD systems in previous studies, although a large number of phage-like particles have been found in methanogenic digesters [9].

AD reactors containing different substrates have revealed the genetic diversity and specificity of viruses [10], but there have been few studies on viral characterization in the anerobic digestion of food waste (ADFW). In fact, environmental variability due to the heterogeneity of FW often destabilizes, ultimately leading to inefficiencies or even failure of the ADFW systems. Several factors can hinder the ADFW. One of these challenges is the high concentration of ammoniacal nitrogen during the rapid degradation of nitrogen-rich organic compounds such as proteins in FW. Both ammonium ions (NH_4_^+^) and free ammonia (NH_3_) can inhibit methanogens and lead to a low methane production [11]. In addition, the rich lipids in FW are readily hydrolyzed to long-chain fatty acids (LCFAs) during the transportation chain, resulting in the inability to remove in the oil extraction unit and thus enter the ADFW reactors. On the one hand, using LCFAs as feed can improve methane productivity. However, the degradation of LCFAs is a non-spontaneous reaction. Therefore, the accumulation of LCFAs can affect cellular mass transfer and trigger ADFW instability [12]. In general, ammonia-rich and LCFA-rich environments are typical scenarios during the practical operation of full-scale ADFW reactors.

Viruses, as parasitic organisms, play a key role in regulating microflora through lysis or lysogen patterns, thereby adjusting the relative abundance of host communities and indirectly reshaping non-target groups by releasing niches [13]. In addition, they also encode the auxiliary metabolic genes (AMGs) in the carbon metabolism [14], the nitrogen cycle [15], and the sulfur metabolism [16], which may affect the metabolic function of the hosts. Previous research regarding AD viruses confirmed that phages are the main biological factor influencing prokaryotic composition and that they control the performance of the AD process [17]. In addition, the phage-host interactions were revealed to be one of the factors shaping microbial communities [18]. Rossi et al. [19] explored the reasons why viruses affect microbial communities from a behavioral perspective. Related studies have provided a preview of the importance of viruses in the microbial communities of AD systems. Nevertheless, as far as can be ascertained, few studies have investigated the behavior of viruses under typical ADFW environmental variability conditions over time and their impact on methanogenesis.

This study aimed to identify the diversity of viruses in ADFW reactors and expand our understanding about the ecological effects of viruses on methanogenesis. Therefore, the existing characteristics of viruses in five full-scale ADFW reactors were investigated. To further explore the behavior of viruses in the ADFW under typical scenarios in industrial applications, a continuous cultivation experiment was performed using lab-scale reactors with ammonium-rich and LCFA-rich environments. The kinetics relationship between viruses and prokaryotes was investigated using a time-series analysis. The effects of viruses on the functional microorganisms associated with methanogenesis was studied from the population and gene perspectives by predicting potential hosts and identifying the AMGs. This study analyzed the ecological role played by viruses and proposed the potential viral mechanisms by which they could affect methanogenic efficiency in ADFW systems.

## Methods

### Sample collection and description of the incubation reactors

Five samples were collected from five mesophilic full-scale continuously stirred tank reactors at the food waste treatment plant in Shanghai, China (referred to LG). Details about the operation of the full-scale reactors and sampling are described in Supplementary Methods S1. The semi-continuous lab-scale cultivation experiment was started in 2-L (actual working volume was 1.8 L) continuously stirred tank reactors with feed ports, discharge ports, and a continuous stirring device at 38.0 ± 0.5 °C (BPC Instruments AB, Lund, Sweden). Mixed slurry samples from the five LG reactors were used as inoculum. The original feedstock was collected from the liquid-like organic slurry entering the full-scale ADFW reactors and stored at 4 °C. Three groups were set up, which were a control group, an ammonium-rich group, and an LCFA-rich group (labeled the C-group, N-group, and L-group, respectively). The cultivation period for the three reactors lasted 126 days and consisted of four stages. During incubation, the organic loading rate (OLR) was increased from 0.5 to 1.3 g-VS/L·day by making four adjustments to the feedstock volume to maintain stable and efficient operation of the reactors. Stage I lasted for 44 days, during which the three reactors were fed with original feedstock and the OLR was adjusted twice from 0.5 to 0.83 g-VS/L·day. Stage II was incubated with an OLR of 0.83 g-VS/L·day until the 92nd day and the feeding strategy was adjusted according the experimental setup. The C-group was fed with original feedstock, whereas the N-group was supplied with extra NH_4_Cl in the original feedstock and the L-group was supplemented with sodium palmitate of 0.1 mmol/L. Stage III and Stage IV operated with OLRs of 1.04 and 1.3 g-VS/L·day, respectively. The N-group maintained a total ammonia nitrogen (TAN) within the range 6000–8000 mg/L by controlling the addition of NH_4_Cl. The concentration of the palmitate added to the L-group was continuously increased to 0.4 mmol/L. Biogas and digestate samples were routinely collected and were used for physicochemical analysis during incubation. A total of 15 samples were taken from the three lab-scale reactors on the 0th, 44th, 92nd, 108th, and 126th days for microbiological testing (Tested samples were termed as C0, C44, C92, C108, C126, N0, N44, N92, N108, N126, L0, L44, L92, L108, L126, where *C* indicates C-group, *N* indicates N-group, *L* indicates L group, and the numeral indicates the operation days) (Details about the lab-scale reactors incubation can be found in Supplementary Methods S2 and daily feed composition is listed in Table S1).

### Physicochemical properties analysis

The biogas volume was measured with a gas meter, and the biogas compositions (CH_4_ and CO_2_) were monitored by a biogas analyzer (Geotech, Great Yarmouth, UK). The pH of the freshly collected samples was immediately measured using a benchtop pH meter (PHS-3BW, Bante Instruments). All samples were centrifuged at 15,000 rpm for 10 min to obtain the supernatant for the determination of soluble chemical oxygen demand (sCOD), total organic carbon (TOC), total nitrogen (TN), TAN, and volatile fatty acids (VFAs). The sCOD and TAN were analyzed using the spectrophotometer (DR3900, HACH, USA) according to the manual of the HACH COD reagent kit (20–1500 mg/L COD, HACH, USA) and the HACH nitrogen-ammonia reagent set with salicylate method (0.01–0.50 mg/L NH_3_-N, HACH, USA), respectively. TOC and TN were determined using a total organic carbon analyzer matching TN unit (TOC-V CPH, Shimadzu, Kyoto, Japan), and the VFAs were determined using a gas chromatography equipped with a flame ionization detector (TRACE 1300, Thermo Fisher Scientific, Waltham, MA, USA) (see details in Supplementary Methods S3).

### DNA extraction, metagenomic sequencing, and assembly

The DNA from five full-scale and 15 lab-scale ADFW samples was extracted using a PowerSoil™ DNA isolation kit (MoBio Laboratories Inc, Carlsbad, CA, USA) according to the manufacturer’s instructions. The extracted DNA was stored at − 40℃ for metagenomic sequencing. Briefly, metagenome libraries were prepared using a NEBNext® UltraTM DNA Library Prep Kit for Illumina (New England Biolabs, Ipswich, MA, USA) according to the manufacturer’s instructions. The libraries were paired-end (2 × 150 bp) and sequenced on Illumina platforms using the PE150 strategy. Metagenome sequencing was undertaken by Novogene Bioinformatics Technology Co., Ltd (Beijing, China). The metagenomic sequencing of each sample yielded an average of 15 Gb of raw reads. First, paired-end metagenomic raw reads were performed using Fastq (v0.19.7) [20] to remove adapter contamination and low-quality bases and then the Read_QC module within metaWRAP (v1.3.2) [21] was used for further trimming. The clean reads from each sample were individually assembled using the ASSEMBLY module within the metaWRAP (v1.3.2) [21] pipeline (choose the metaSPAdes method with kmers of 21, 33, and 55). The final assembled contigs shorter than 1000 bp were removed (see details in Supplementary Methods S4 and S5).

### Metagenomic binning and taxonomy of prokaryotes

The assembled contigs from the 15 lab-scale reactor samples were binned based on three methods: maxbin2, metabat1, and metabat2, which were performed by the Binning module. The final bin sets were consolidated predicted bins that had been obtained using the Bin_refinement module within metaWRAP (v1.3.2) [21]. The quality of the bins was assessed by CheckM (v1.1.3) [22], including completeness and contamination. In general, low-quality bins where completeness was lower than 50% or contamination was higher than 10% were discarded. The filtered bins were de-replicated at 95% average nucleotide identity (ANI) to produce metagenome-assembled genomes (MAGs) using dRep (v3.2.2) [23]. Taxonomic classification of the MAGs was assigned using GTDB-TK (v1.7.0) [24] based on the Genome Taxonomy Database (05-RS95 release). The resulting maximum-likelihood phylogenetic tree was visualized and refined using iTOL (v6.5.8) [25] (see details in Supplementary Methods S6).

### Viral contigs identification and taxonomic assignments

A total of three accepted tools were adopted to recover viral contigs, which were Virsorter2 [26], DeepVirfinder [27], and CheckV (v0.8.1) [28]. Only contigs longer than 1.5 kb were retained on following criteria: (1) Virsoter2 score ≥ 0.95; (2) DeepVirfinder score ≥ 0.9 and *p* < 0.05; (3) identified by Virsorter2 and DeepVirfinder score ≥ 0.7 and *p* < 0.05. Contigs that met any of the above three criteria were retained and inputted into CheckV (v0.8.1) [28] for quality evaluation. Any contigs identified as “Not-determined” by CheckV (v0.8.1) [28] were further filtered. The open reading frames (ORFs) of these contigs were identified by Prodigal (v2.6.3) [29], and the protein sequences were annotated to the NCBI RefSeq viral protein database (https://ftp.ncbi.nlm.nih.gov/refseq/release/viral) using Diamond (v2.0.14.152) [30] with a e-value of 1e^−5^. Those contigs with no gene hit were removed. All remaining contigs were grouped into viral operational taxonomic units (vOTUs) if they shared ≥ 95% nucleotide identity using CD-HIT (v4.8.1) [31]. For each vOTU, the ORFs predicted by Prodigal (v2.6.3) [29] were aligned against the NCBI Refseq viral proteins database using BLASTp (e-value of 1e^−5^) [32]. The taxonomic affiliations of each vOTU were predicted using the lowest common ancestor (LCA) algorithm within Taxonkit (v0.10.1) [33] to calculate the LCA of all hit genes in one vOTU. The vOTUs were finally classified using the NCBI taxonomy naming system [34]. The vOTUs that had no specific affiliation to any known taxa were classified as “Unknown”.

### Virus network construction

A gene-sharing network was used for correlation exploration. All ORFs predicted by Prodigal (v2.6.3) [29] were uploaded to vConTACT2 [35] to produce viral clusters (VCs). Diamond was used to detect protein–protein similarity method, and the protein clusters were generated by the Markov Cluster Algorithm (MCL). VCs were generated by ClusterONE [36]. The gene-sharing network results were visualized in Cytoscape (v3.8.2) [37]. To compare the similarities and differences between the data set produced by our study and other broader habitats, viral sequences for three engineered ecosystems were downloaded from IMG/VR database v3.0 [38]. The three ecosystems were (i) wastewater (*n* = 7452; *n*: the number of viral sequences); (ii) bioreactor (*n* = 6513); and (iii) lab enrichment (*n* = 1090). Each ecosystem contained both anaerobic (*n* = 5163, 5911, 736) and aerobic (*n* = 2289, 602, 354) subclasses.

### Host prediction

A total of 169 MAGs were used to predict “in situ” hosts for the vOTUs derived from same sample. The linkages of vOTUs and prokaryotic genomes were performed using CRISPR spacer matching according to Martínez Arbas et al. [39], with minor modification. In brief, identification of protospacer-containing contigs workflow was applicated for vOTUs. The final virus-host linkages were defined by the spacer that co-occurred with its assigned MAGs, which then targeted vOTUs in the same sample. These interrelations were deemed to be more active.

### Gene analysis

The gene and protein sequences of each MAG were analyzed using EnrichM (v0.6.4) (https://github.com/geronimp/enrichM) based on Kyoto Encyclopedia of Genes and Genome (KEGG) Orthogroups (KOs) for metabolic reconstruction. For identified vOTUs, CheckV (v0.8.1) [28] was used to remove host contamination. The clean vOTUs were called ORF sequences by Prodigal (v2.6.3) [29], and the ORF sets were further annotated using EnrichM (v0.6.4) for metabolic pathway analysis.

### Relative abundance profiles

The relative abundances of the viruses and prokaryotes were expressed in the form of transcripts per kilobase of exon model per million mapped reads (TPM) [40]. To estimate the TPM values of each vOTU and MAG, clean reads from each sample were mapped to a reference database of vOTUs or the 169 MAGs using CoverM (v0.6.1) (https://github.com/wwood/CoverM) with the contig or genome mode. Bwa-mem was chosen as the method for calculating coverage. The low-quality alignments were removed (reads identity ≤ 95% and aligned percent ≤ 75%), and the relative abundances of the taxa in the vOTUs and MAGs were counted by summing up all the relative abundances in the same taxon.

### Statistical analyses

All statistical analyses were performed in R version 4.0.5. The alpha diversities of the virus and prokaryote communities were calculated using the Vegan (v2.5–7) package [41]; a principal coordinate analysis (PCoA) was performed based on Bray–Curtis dissimilarities generated from the relative abundances of viruses and prokaryotes; and an analysis of similarity (ANOSIM) was used to verify the different time point samples, which was performed by the Vegan (v2.5–7) package [41]. Vegan (v2.5–7) [41] was also used to calculate Pearson correlations between viruses and their predicted host based on their relative abundance in each sample. A multiple regression matrix (MRM) analysis was performed to test the correlation between microbial communities and abiotic factors using Vegan (v2.5–7) [41] and Ecodist packages [42].

### Availability of data and materials

All raw reads associated with metagenomic sequencing in this study have been submitted to the NCBI Sequence Read Archive under the project ID PRJNA867664.

## Results

### Industrial ADFW systems contained novel and diverse virus pools

From the five full-scale ADFW reactor samples, 5036 vOTUs were recovered using the identification pipeline with one vOTU greater than 300 kb in length that might be assigned to a huge virus [43]. According to the estimation using CheckV (v0.8.1) [28], 93 vOTUs with completeness higher than 50% were assessed as either complete, high-quality, or medium-quality (Table S2). The classification analysis of 5036 vOTUs found that 46.8% of the viral community could not be attributed to any known family. *Siphoviridae* (32.7%), *Myoviridae* (11.9%), and *Podoviridae* (3.0%) were the dominant classifiable families, accounting for 47.6% of the total composition (Figure S1 and Table S3).

**Fig. 1:**
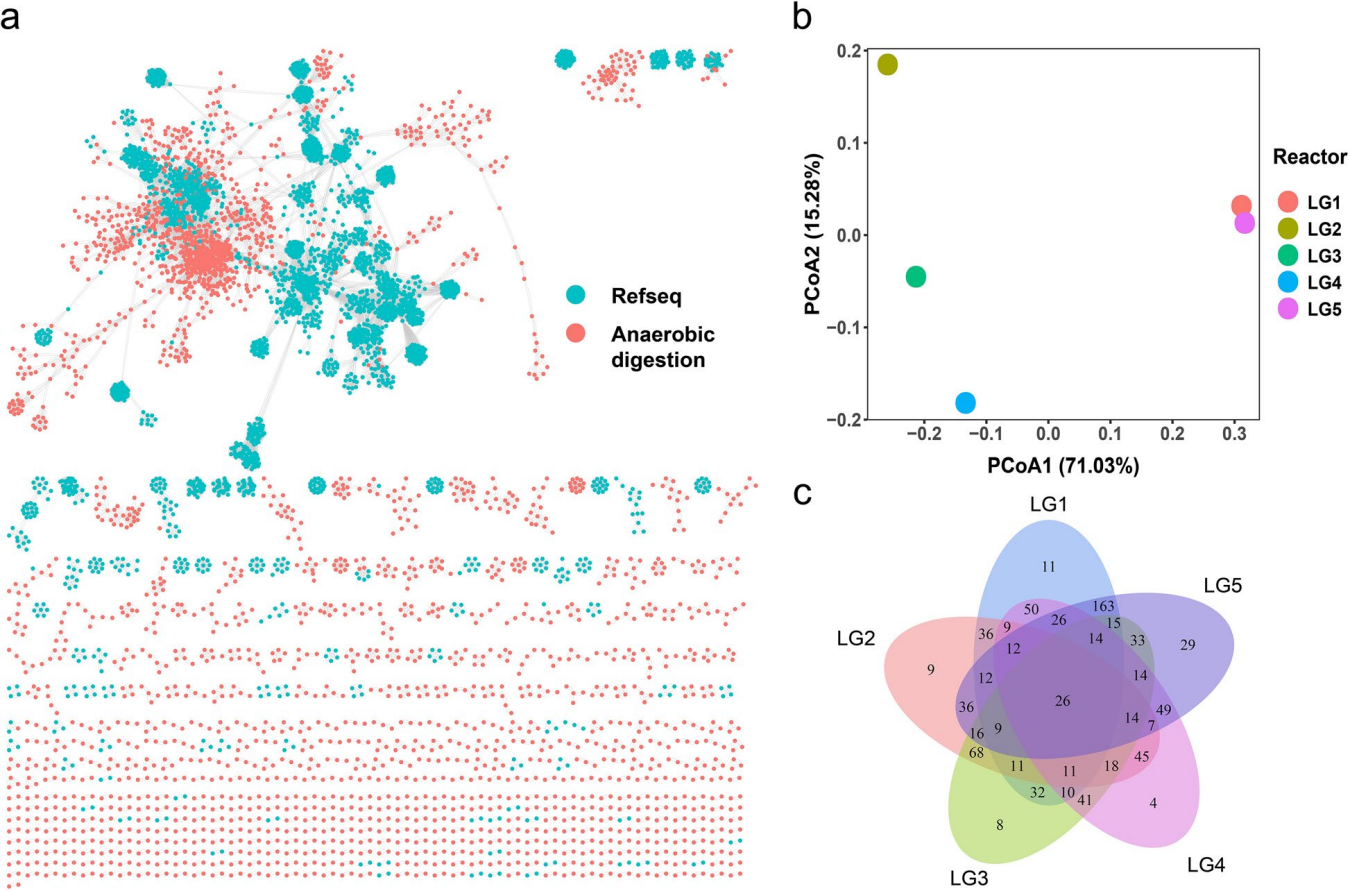
Characteristics of ADFW viruses in full-scale reactors. **a** Gene-sharing network of viral sequences in full-scale reactors and NCBI Refseq viral genomes. Circles represent viral sequences and edges connecting the two points indicate proteins sharing. **b** The PCoA analysis based on the Bray–Curtis dissimilarity matrix was calculated from the TPM values of the vOTUs to compare the distances between each reactor. **c** Venn diagram indicates the shared protein clusters among the five full-scale reactors based on the vConTACT2

A gene-sharing network was constructed by vConTACT2 [35] to investigate the relationship between ADFW vOTUs and the NCBI reference viral genomes. Consequently, 1278 VCs were grouped at approximately the genus level, of which only 14 (1.10%) VCs were shared with the known viral genomes in NCBI Refseq (v.201), with the majority of VCs (64.48%) were separate aggregations of ADFW viruses (Fig. 1a and Table S4). These findings prompted further research comparing ADFW viruses to those in broader habitats. Partial viral sequences in the IMG/VR database v3.0 [38] were used to explore the co-occurrence between ADFW viruses and other specific ecosystems. A total of 15,055 viral sequences were used to construct the gene-sharing network. The results showed that the vOTUs identified in this study were shared among all other ecosystems except for the aerobic subclass of the bioreactor. There was a considerable number of shared VCs between ADFW and the anaerobic subclass compared to the aerobic subclass (Figure S2).

Further analysis of the relevancies among the five full-scale reactor samples showed that these samples were distanced from each other, with only one pair: LG1 and LG5, having a high similarity (Fig. 1b). As shown in the gene-sharing network (Fig. 1a), ADFW vOTUs were clustered into 838 VCs, of which only 26 (3.1%) VCs were detected in all five reactors and 61 (7.3%) VCs were unique. However, the vast majority of VCs were shared among more than two reactors (Fig. 1c). In general, reactors in the same plant had similar feedstock and operating conditions, which meant that the ADFW viral communities showed commonality, but also variability due to the individuality of each reactor.

### Performance during operation of the lab-scale reactors

**Fig. 2:**
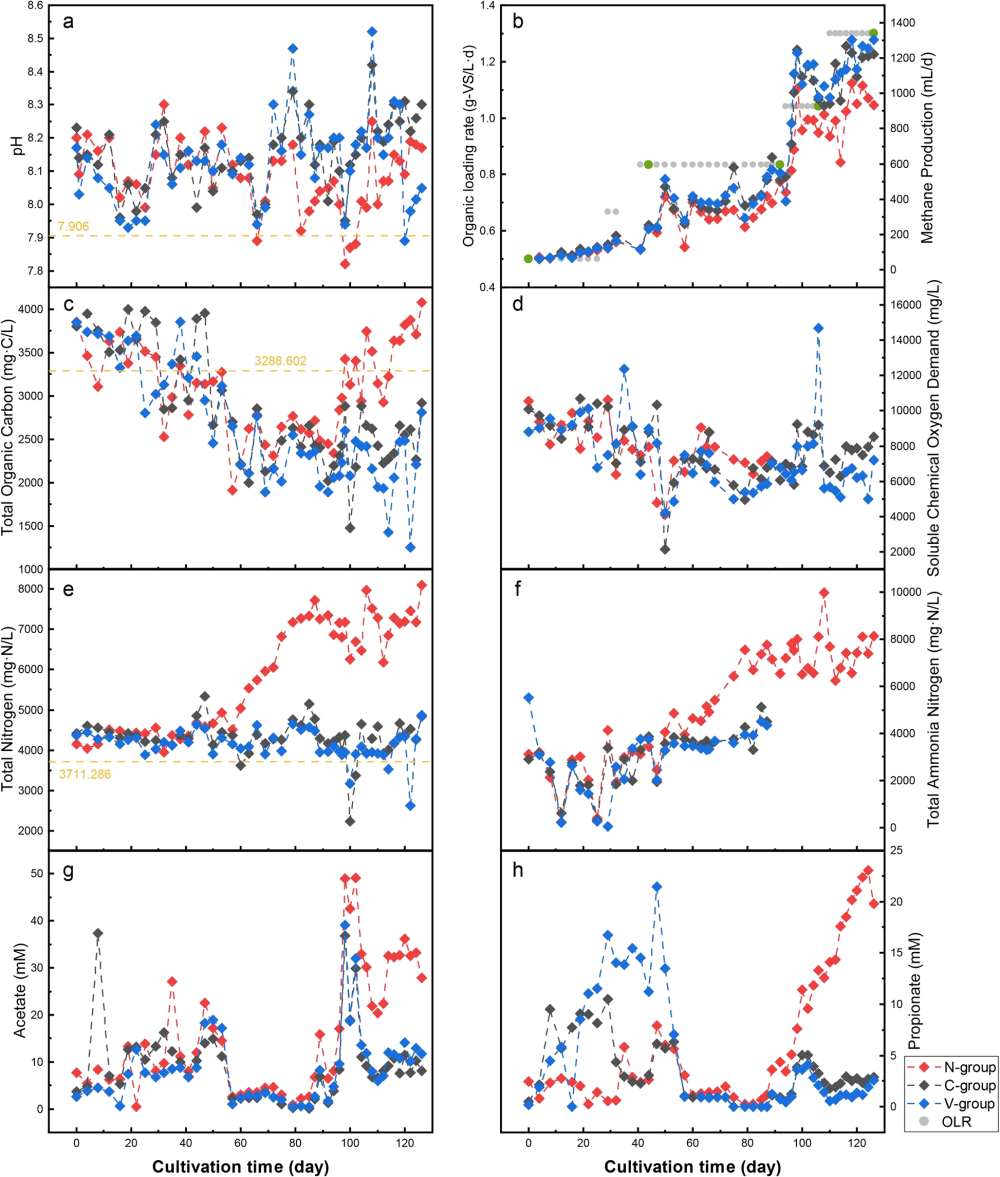
Dynamics of physicochemical parameters during cultivation in the three lab-scale reactors. **a** pH; **b** methane production, green dots indicate microbial sampling points; **c** total organic carbon (TOC); **d** soluble chemical oxygen demand (sCOD); **e** total nitrogen (TN); **f** total ammonia nitrogen (TAN); **g** acetate; and **h** propionate. Orange dashed lines indicate the average values for the five full-scale reactor samples

As shown in Fig. 2, the OLR in Stage I was artificially raised twice from 0.5 to 0.83 g-VS/L·day by adjusting the feedstock volume. During this period, the three reactors operated smoothly with similar physicochemical properties. They maintained the pH between 7.93 and 8.30 and the TN between 3881 and 4854 mg/L, which were slightly above the average values for pH and TN in full-scale reactors. The TOC and sCOD values showed a downward trend, whereas acetate concentration increased. The methane production increased from 65.20 (average on day 0) to 240.07 mL/day (average on 44th day), an almost fourfold increase. The results indicated that the organic matter during Stage I rapidly degraded and that the reactors were operating efficiently with a potential to increase the organic load of the operation.

The introduction of extra ammonium in Stage II resulted in a rapid increase in TN and TAN concentrations in the N-group, with TN rising from 4585 (47th day) to 7347 mg/L (92nd day) and TAN rising from 2440 (47th day) to 6526 mg/L (92nd day). At this time, the TOC, sCOD, acetate, and propionate concentrations in the N-group were slightly higher than those in the C-group, but the pH was slightly, but not significantly lower. However, when compared to the C-group, the maximum difference in methane production reached 258.85 mL/d (89th day), indicating that the addition of ammonium reduced methane production efficiency to some extent. The L-group was not significantly different from the C-group in Stage II, with similar fluctuations in physicochemical parameters and methane production.

In Stages III and IV, the acetate concentration in the C-group was higher than that of Stage II, but TOC and sCOD were comparable to Stage II and the methane production substantially increased, suggesting that the increased OLR allowed more organic matter to be degraded to methane. The TN and TAN levels in the N-group ranged from 6000 to 8000 mg/L and TOC was significantly higher than in the C-group, indicating that organic matter acidification was restricted. Acetate was observed to accumulate and the propionate concentration continued to rise. Methane production of the N-group in Stages III and IV was approximately 4 L less than that of the C-group, indicating that methanogenesis was also impeded. The TOC and sCOD levels were slightly lower in the L-group than in the C-group, but degraded organic matter did not significantly increase the production of methane (details about the physicochemical parameters can be found in Table S5).

### Virus and prokaryote dynamics under ammonium-rich or LCFA-rich conditions

The three lab-scale reactors were used to further investigate the behavioral characteristics of the viruses in ADFW under ammonium-rich or LCFA-rich conditions over time. A total of 13,691 vOTUs were eventually recovered from the 15 metagenomes (Table S6) and two-fifths of the viral community could not be attributed to a known annotation at the family level (Table S7). *Siphoviridae* was the most dominant family in all samples (35.1%), while *Myoviridae* (14.9%) and *Podoviridae* (2.8%) had high relative abundances (Figure S3), which was similar to the full-scale reactors. The high- or medium-quality binned genomes were demonstrated by the 169 MAGs used to access the characteristics of prokaryotes (Table S8).

**Fig. 3:**
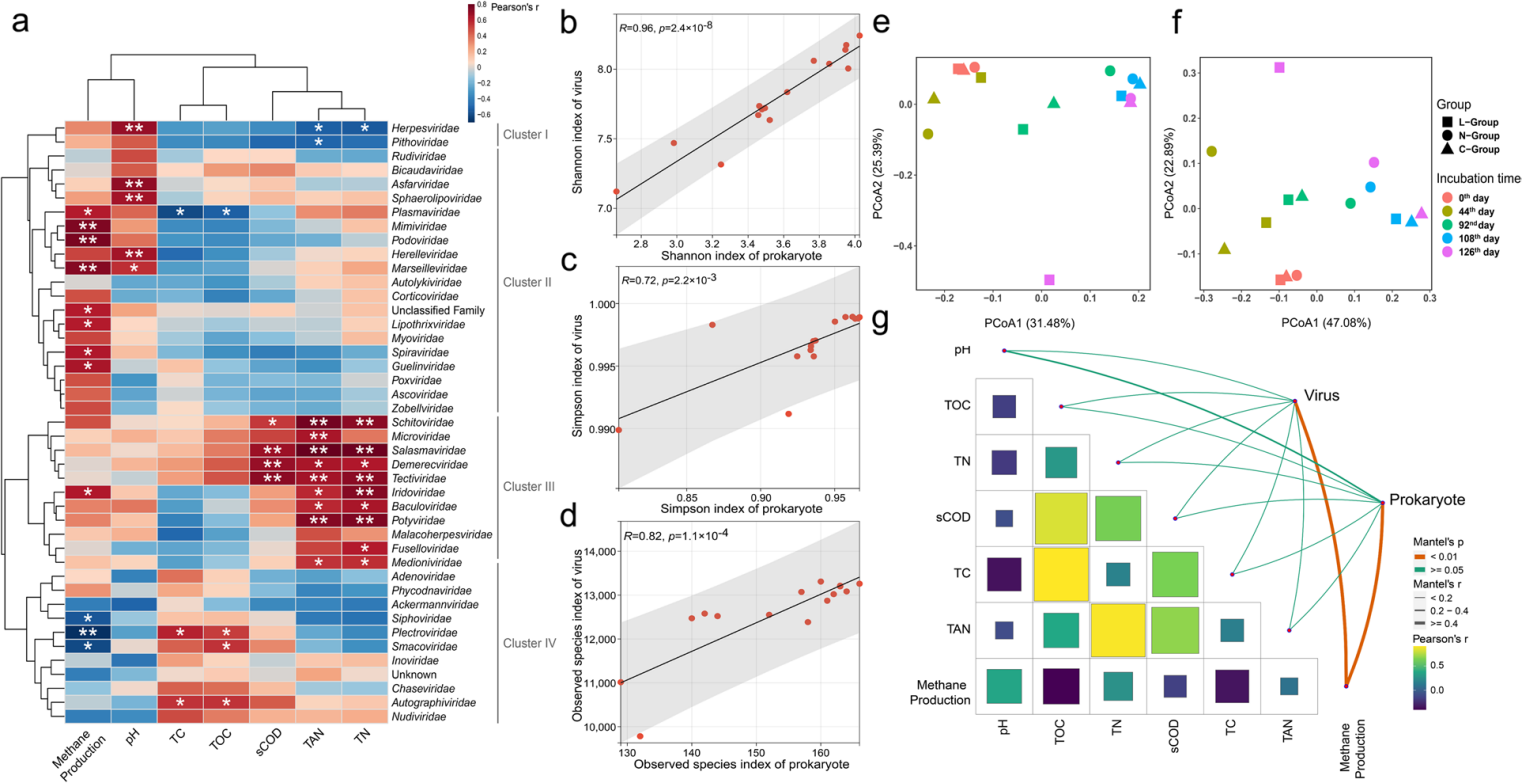
Dynamics of viruses and prokaryotes during cultivation. **a** Relationships between viral taxa and physicochemical parameters were calculated by Pearson correlation. Asterisks show significance, where *p* < 0.05 is indicated by^*^ and *p* < 0.01 is indicated by ^**^. Pearson’s correlations for **b** Shannon indexes, **c** Simpson indexes, and **d** observed species indexes between viruses and prokaryotes. PCoA of **e** viruses and **f** prokaryotes derived from Bray-Cutis dissimilarities was performed using the TPM values. The ANOSIM test was applied to assess the differences among sample dates. **g** Pairwise comparisons of the biotic and physicochemical parameters using the Mantel test. Heatmap indicates Pearson correlations between pairwise abiotic factors

The dependence of viral populations on different factors was assessed using Pearson correlations to reveal the relationships between viral taxa and abiotic factors. The results showed that the majority of taxa were affected by different physicochemical properties (Fig. 3a and Table S9). Based on the clustering results, viral families were divided into four clusters. Two viral families in Cluster I were negatively correlated with either TAN or TN. However, all viral families in Cluster III, except *Malacoherpesviridae*, were positively correlated with TAN or TN and four of them: *Salasmaviridae*, *Schitoviridae*, *Tectiviridae*, and *Potyviridae*, were significantly correlated with both TAN and TN. In Stages III and IV, the relative abundances of the *Salasmaviridae*, *Schitoviridae*, *Tectiviridae*, and *Potyviridae* in the N-group were 3.83, 4.04, 2.23, and 1.98-fold higher than in the C-group, respectively (calculated from the mean abundance values on the 92nd, 108th, and 126th days). The results indicated that members of the *Salasmaviridae*, *Schitoviridae*, *Tectiviridae*, and *Potyviridae* could potentially become acclimatized to high ammonium conditions. The majority of the viral families in Cluster II showed positive correlations with methane production and pH, with *Mimiviridae*, *Podoviridae*, and *Marseilleviridae* significantly correlated with methane production. However, the *Plectroviridae* members in Cluster IV showed a negative correlation. The dual characteristics revealed the variable responses of different viral taxa to methane production.

The correlations between viral and prokaryotic community diversities were examined to verify the virus-prokaryote dynamics. Specifically, the Shannon diversity (*R* = 0.95, *p* = 2.4 × 10^−8^; Fig. 3b) and Simpson diversity (*R* = 0.72, *p* = 0.0023; Fig. 3c) indexes for prokaryotes and viruses were significantly correlated. In addition, there was a correlation with the observed species index (*R* = 0.83, *p* = 1.1 × 10^−4^; Fig. 3d) (details about the diversity indexes in Table S10). The PCoA analysis was used to reveal the clustering patterns for the viral community (ANOSIM, *R* = 0.5674, *p* = 0.002; Fig. 3e) and prokaryotic community (ANOSIM, *R* = 0.7422, *p* = 0.001; Fig. 3f) across the time series. The results showed that the microbial community structure during the incubating process was significantly different from the original community structure, especially L126 (Fig. 3e and Fig. 3f). The microbial community of the N-group converged after adding NH_4_Cl in feedstock (92nd, 108th, and 126th days). The microbial community in the C-group showed convergence in one direction as the organic loading increased and gathered together when the OLR reached more than 1 g-VS/L·day (108th and 126th days). Meanwhile, the L-group microbial community was not significantly different to the C-group during the initial stage of palmitate addition, but by the 126th day, it showed a clear distance from all other samples. However, when the virus and prokaryote communities were compared, congruous dynamic changes could be observed during the incubating process.

Mantel test was performed to evaluate the factors driving viral and prokaryote community structure (Fig. 3g and Table S11). Methane production was the stronger correlate for both viral and prokaryotic community dissimilarities. Combining the results for methane production and the PCoA analysis showed that the microbial community developed in one direction as methane production increased over time. Furthermore, the results of MRM showed that viruses explained 85.4% of prokaryotic community composition, much higher than the power of abiotic factors (42.96% for pH, TOC, TAN, sCOD, and methane production) (Table S12). In general, microbial community structure can be influenced by multiple factors. Methane production, as the superficial characteristics of microbial synergy, was significantly correlated with viral and prokaryotic community dynamics. However, viruses have a much higher impact on prokaryotes than abiotic factors, so the shaping of prokaryotic communities by viruses may be underestimated.

### Overview of prokaryotic communities associated with methanogenesis in lab-scale reactors

A total of 169 MAGs containing 159 bacterial and 10 archaeal MAGs were used to evaluate the prokaryotic succession patterns under different feeding conditions. Within the bacteria, the dominant members were *Firmicutes* (59.9%), *Bacteroidota* (8.2%), *Chloroflexota* (7.8%), and *Cloacimonadota* (6.1%) (Table S8). They were the common hydrolytic acidifying bacteria in ADFW and accounted for 82% of total composition. For archaeal lineages, members were mainly affiliated with *Methanobacterita* (5.0%) and *Halobacteriota* (4.8%) (Figure S4). Archaea produces methane by consuming acetate (acetoclastic methanogenesis, AM), methylated compounds (methylotrophic methanogenesis, MM), and H_2_ and CO_2_ (hydrogenotrophic methanogenesis, HM) [18]. For the HM pathway, the syntrophy of syntrophic acetate-oxidizing bacteria (SAOB) is necessary. SAOB employs the oxidative Wood-Ljungdahl (WL) pathway to convert acetate into H_2_ and CO_2_ for hydrogenotrophic archaea consuming [44], called syntrophic acetate oxidation coupled with hydrogenotrophic methanogenesis (SAO-HM). On this basis, the interesting findings were observed that the functional genes encoding a complete or nearly-complete oxidative WL pathway can be reconstructed in four high-quality MAGs (L92bin.31, L44bin.114, N92bin.52, N126bin.29) (Figure S5 and Table S13). All four members were classified as *Firmicutes*, including two known SAOB *Syntrophaceticus schinkii* (N92bin.52) [45] and *Tepidanaerobacter acetatoxydans* (L44bin.114) [46], while the other two MAGs (L92bin.31, N126bin.29) were assigned to the DTU022 order.

**Fig. 4:**
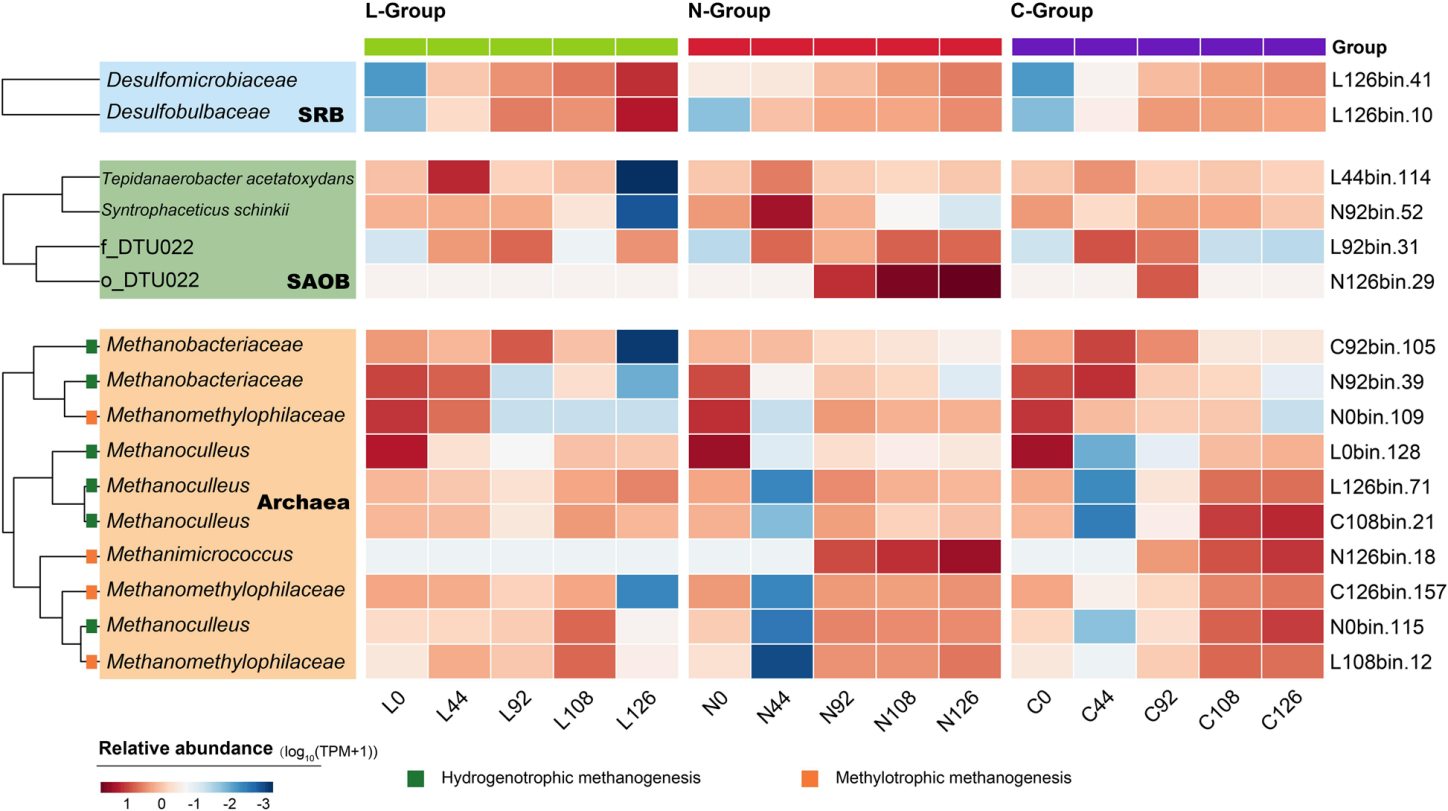
Dynamics of key functional microbial communities. Changes in the compositions of prokaryotes related to methanogenesis during the incubation period, including SRB, SAOB, and archaea. Relative abundance values were logarithmically calculated by Log_10_ (TPM + 1) and standardized using *Z*-score for visualization

Regarding the impact of LCFAs on methanogenesis pathway, the relative abundance of archaea in the L-group decreased compared to that in the C-group (Fig. 4 and Table S8). However, the proportion of *Methanoculleus* members (L126bin.71, C108bin.21, N0bin.115, and L0bin.128) in the total archaea composition increased from 33.4% (0th day) to 97.1% (126th day). Similarly, a clear shift of L92bin.31 was observed with its relative abundance ranging from 2.21% (0th day) to 5.82% (126th day), eventually accounting for 74.4% of all SAOB abundance. Other archaea members seemed not to be actively working under the LCFA-rich condition, as shown the decline abundance of *Methanobacteriaceae* and the consistently low abundance of *Methanomethylophilaceae*. These results showed that *Methanoculleus* dominated HM pathway became the dominant pathway during the late stages. In addition, two MAGs (L126bin.41 and L126bin.10) assigned to the phylum *Desulfobacterota* were considered to be typical sulfate-reducing bacteria (SRB) that could undertake sulfate reduction coupling to convert LCFAs into CO_2_ and H_2_ [47, 48]. Both of them significantly increased by more than threefold in Stage IV. Therefore, hydrogenotrophic methanogens and SRB shared the responsibility for the metabolism of substances in the LCFA-rich environment.

For the N-group, the relative abundance of DTU022 members was more than 3.55-fold that of the C-group in Stages III and IV, despite the other two SAOB did not show activity in response to elevated ammonium concentrations. Previously, related research has confirmed the dominance of SAOB under high ammonium condition [49, 50]. The trend for L0bin.128, which was classified as *Methanimicrococcus sp012518265*, was significant. L0bin.128 was not detected at the initial stage but started to grow at Stage II in the N and C-groups. Moreover, the relative abundances of L0bin.128 in the N-group were 6.12-, 2.08-, and 3.71-fold higher than that in the C-group at the 92nd, 108th, and 126th days, demonstrating its higher growth rate in the ammonium-rich environment. Members of *Methanimicrococcus* have been reported to perform the methanogenesis pathways using methanol or methylamine [51]. The addition of ammonium may facilitate the methanogenesis pathway associated with *Methanimicrococcus sp012518265* consumption of methylamine. Overall, the results demonstrated the active roles played by SAOB and methylotrophic methanogen during methanogenesis in an ammonium-rich atmosphere.

The screening of the broader prokaryotic communities was oriented toward the bacteria and archaea that affected the methanogenesis pathway. In general, SAOB and SRB in the bacterial domain and methanogens in the archaeal domain were considered to be the significant prokaryotic players during the cultivation period.

### Viruses infect functional microorganisms related to methanogenesis

**Fig. 5:**
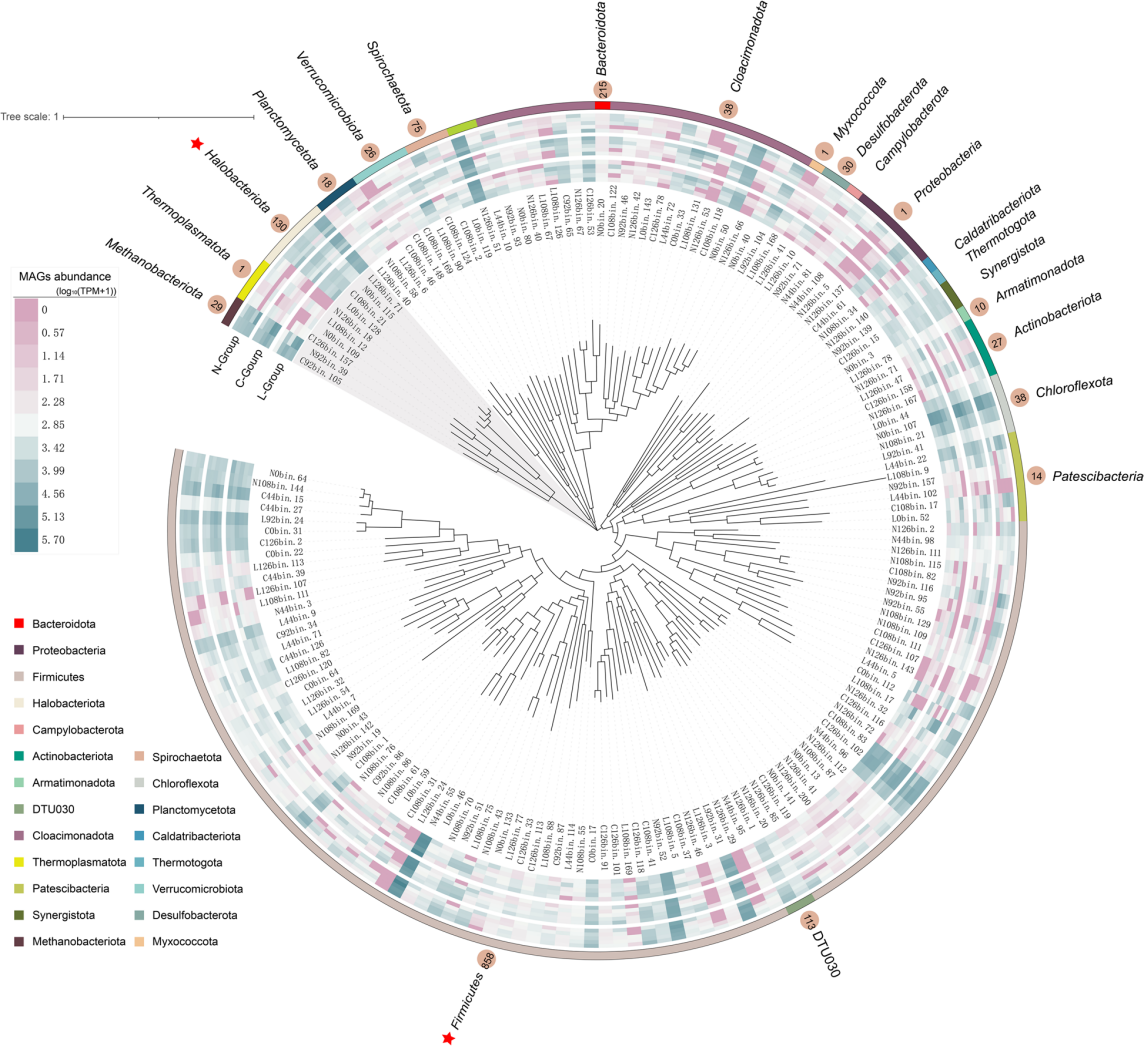
Virus-host linkages in ADFW systems. A total of 169 MAGs were used to construct the maximum-likelihood phylogenetic trees. The relative abundance of each MAG in the three groups is shown in the inner circle, from inside to outside on days 0, 44, 92, 108, and 126. Black labels represent MAG taxonomic assignment at the phylum level and circles show the number of vOTUs with predicted hosts in each phylum. The bacterial and archaeal phylum linked to the most vOTUs are marked with red stars

CRISPR-Cas spacer matching was used to predict hosts for 13,691 vOTUs. A total of 1631 spacers that assigned at least one MAG and targeted at least one vOTU in the same sample were retained. Based on this, 1182 vOTUs (8.63%) were linked to a putative host, which was 0.95–28.26% of the vOTUs in each sample (Table S14). These predicted hosts spanned a wide range of bacteria and archaea, with 14 bacterial and three archaeal phyla. Within the bacterial domain, 858 vOTUs were linked to *Firmicutes*, which was higher than that of the other bacterial phyla. *Bacteroidota* (*n* = 215; *n*: the number of vOTUs linked to a phylum) and DTU030 (*n* = 113) also contained a large number of predicted hosts (Fig. 5). Among archaea, a considerable proportion of vOTUs infected *Halobacteriota* (*n* = 130), and *Methanobacteriota* (*n* = 29) (Fig. 5). Among these, the vast majority of phylum have not been previously reported as being infected by viruses. Most of the 1182 vOTUs were assigned to a narrow host range. However, 321 vOTUs exhibited the potential to infect more than one host. In particular, 68 vOTUs can infect bacteria and archaea simultaneously, suggesting the existence of viruses that can infect different domains (Figure S6).

The relationships between viruses and key functional microorganisms were also investigated. There were 10 and 20 vOTUs identified as being potentially able to infest *Desulfomicrobium* and *Desulfobulbus*, respectively. The vast majority of vOTUs (90 and 75%, respectively) associated with the two hosts showed a significant positive correlation (*p* < 0.001; Figure S7a), which indicated that viruses may indirectly regulate LCFAs metabolism by affecting SRB. After further exploring the characteristics of the infested SAOB viruses, the results showed that each SAOB was infected by several vOTUs, except N126bin.29 (Figure S7b). In contrast to the SRB infection characteristics, only a small proportion of vOTUs showed a significant positive correlation with their SAOB hosts. Only 23.9% of vOTUs displayed a significant positive correlation (*p* < 0.01) for L92bin.31. The proportions were 39% for L44bin.114 and 0% for N92bin.52. However, if the relative abundances of all viruses infecting the same host were added together to calculate the correlation with their host, the host-virus pair interactions showed stronger correlations (*R* = 0.66, *p* = 0.0073 for L92bin.31; *R* = 0.71, *p* = 0.0029 for L44bin.114; and *R* = 0.56, *p* = 0.029 for N92bin.52; Figure S8). The results showed that the viruses infecting SAOB have some more complicated internal mechanisms that may jointly regulate the population structure of SAOB.

With regard to the archaea (Figure S7c), no corresponding vOTU was matched to *Methanimicrococcus sp012518265* and one vOTU was associated with N0bin.115, which belonged to *Methanomethylophilaceae*. Two MAGs (N92bin.39, C92bin.105) assigned to *Methanobacteriaceae* were matched to 28 and 1 vOTUs, respectively. In comparison, four *Methanoculleus* members (L126bin.71, C108bin.21, N0bin.115, L0bin.128) were linked to a number of vOTUs (25, 41, 30, and 34, respectively), which suggested that *Methanoculleus* can be infected by multiple viruses. *Methanoculleus* is the typical hydrogenotrophic methanogen that has a strong tolerance to adverse environments [49]. Multiple viral infestations may boost *Methanoculleus* tolerance in adverse environments. The correlations between archaeal viruses and their hosts showed that most matches were not significant, which may be due to the slow growth rate of methanogens and their susceptibility to external environmental factors. However, there was a particular finding that a large proportion of viruses infected with N92bin.39 (82.14%) and N0bin.115 (53.3%) showed the opposite trend to their hosts (*R* < 0). Therefore, in the archaeal domain, a fraction of viruses was negatively correlated toward their hosts, which may affect competition among methanogens to become the dominant flora (Details information on the characteristics of viruses that infect functional microorganisms associated with methanogenesis can be found in Table S15).

### Viruses that encoded extensive auxiliary metabolic genes

Having demonstrated the profile of viral effects on methanogenic-related functional microorganisms, the functions of viruses in ADFW systems were further investigated using the gene profiles. Viral ORFs annotated using EnrichM were considered to be the broad AMGs that refer to all metabolic genes [52]. Overall, ADFW viruses tended to encode AMGs for substance metabolism, including carbohydrates, energy, lipids, nucleotides, and amino acids (Table S16), demonstrating the high diversity of AMGs in ADFW systems.

The degradation of LCFAs is the rate-limiting step in ADFW systems and is usually accomplished by β-oxidation [53]. Long-chain acyl-CoA synthetase (ACSL) was found to be encoded by three vOTUs that activate LCFAs to form acyl-CoAs. The acyl-CoAs are key intermediates in β-oxidation and play an important role in downstream metabolism [54]. Consequently, results revealed the potential role of the virus-encoded AMGs in the key steps associated with LCFA degradation. Several AMGs were found to be associated with sulfur metabolism. A total of 13 vOTUs encoded cysH, which has been found in viral sequences from various ecosystems and facilitates assimilatory sulfate reduction (ASR) in the host [55–57]. In addition, sulfate adenylyltransferase (sat) within AMGs was observed to be responsible for adenosine 5-phosphosulfate (APS) reduction, which is the final step in the sulfide oxidation reaction during dissimilatory sulfate reduction (DSR).

**Fig. 6:**
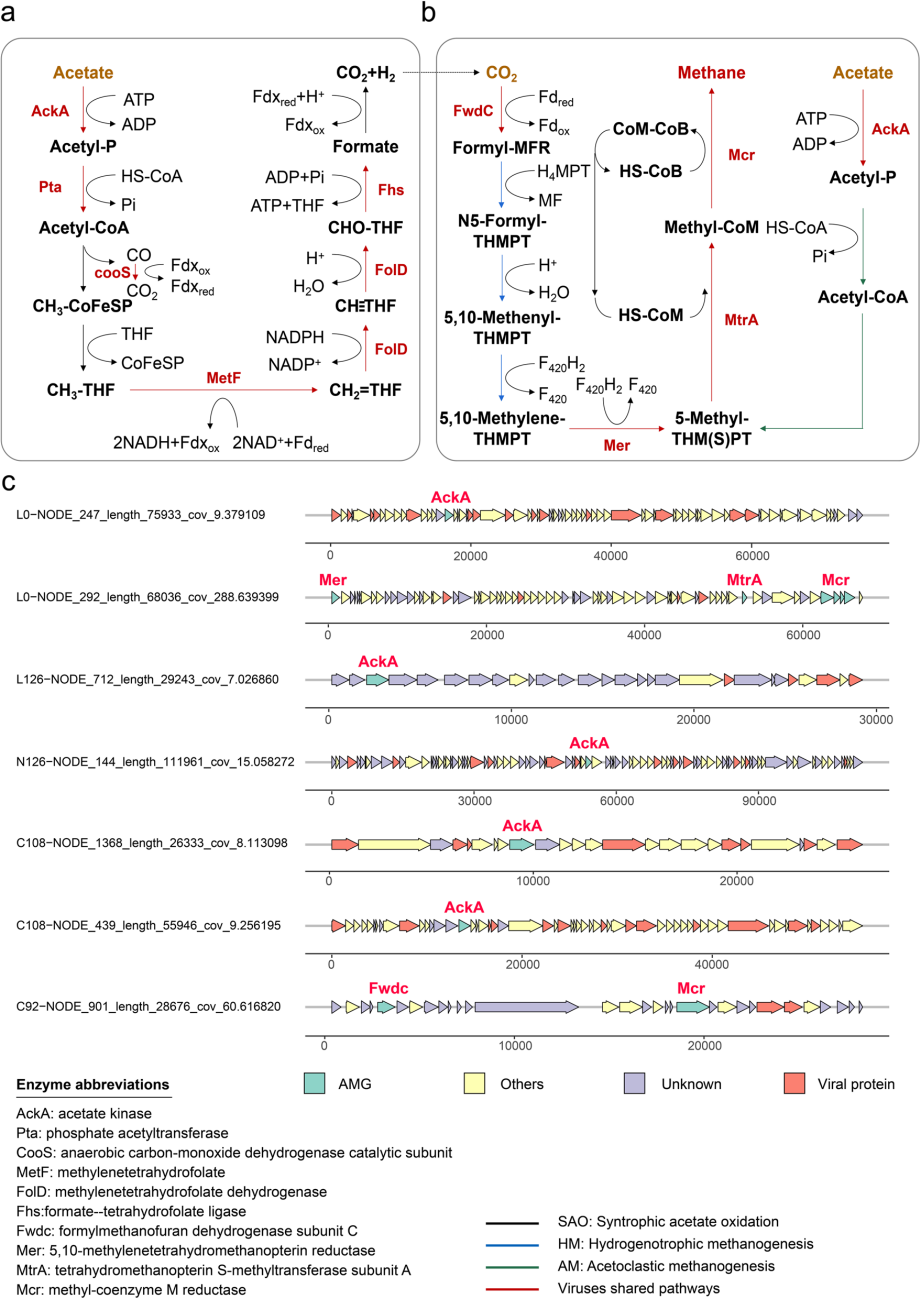
Genomic analyses of virus-encoded methanogenic-related genes. The representative metabolic pathways related to methanogenesis involved in virus-encoded enzymes, AM **b** and SAO-HM **a, b** pathways. **c** Genome map of seven contigs containing methanogenic-related genes

Broadly speaking, 40 AMGs were potentially associated with methanogenesis pathways, of which 37 were labeled as carbon fixation pathways in KEGG. Therefore, the impacts of virus-encoded AMGs on methanogenesis in ADFW systems were further investigated by studying the two major pathways that convert acetate to methane, AM and SAO-HM (Fig. 6a,b and Table S17). For AM pathway, viral members encoded a near-complete list of genes that were involved in the conversion of acetate to methane. The exceptions were the genes encoding phosphate acetyltransferase (Pta) and ACDS, which catalyzes the activation of acetyl-P to 5,10-methyl-THMPT, a key precursor for methanogenesis in both AM and SAO-HM pathways. Although the viruses lacked a complete pathway to convert acetate into 5,10-methyl-THMPT in AM pathway, the genes encoding acetate kinase (AckA) to transformed acetate into acetyl-P were found in five vOTUs (Fig. 6c), which suggested that viruses may contribute to the conversion of acetate to important intermediates. On the other hand, HM pathway produces methane with H_2_ and CO_2_ as substrates and two steps were activated in the transformation of CO_2_ to 5-methyl-THMPT. Two vOTUs could catalyze the first step from CO_2_ to formyl-MFR by formylmethanofuran dehydrogenase (fwdC) and activate the conversion of 5,10-methylene-THMPT to 5-methyl-THMPT using 5,10-methylenetetrahydromethanopterin reductase (mer) (Fig. 6c). In the shared steps of two methanogenesis pathways, viruses encoded the corresponding enzymes to accomplish the reaction from 5,10-methyl-THMPT to methane. The oxidative WL pathway is generally considered to be constructed by SAOB. However, enzymes by viruses were involved in the conversion of acetate to acetyl-CoA. In addition, there were corresponding enzymes that participated in the conversion of CH_3_-THF to formate, which is the main step in the WL pathway. The results from this study demonstrated that viruses had the potential to assist the host in most steps of the WL pathway.

## Discussion

Based on the analysis of metagenomes, this study confirmed that there were a large number of viral sequences in full-scale ADFW reactors and the results significantly expanded the ADFW virome (Fig. 1a and Table S4). Viruses involved in ADFW systems were novel compared to NCBI viral Refseq, possibly because the complete genomes for a majority of viruses have not been obtained (11,607 complete viral genomes in NCBI viral Refseq as of June 2022, https://www.ncbi.nlm.nih.gov/genomes/GenomesGroup.cgi?taxid=10239). In addition, previous studies on viruses focused more on natural ecosystems [5, 7, 58] and human health [59], resulting in a certain bias in the reference database. Unlike natural environments, engineered ecosystems are bio-enhanced facilities with artificial interventions whose microbial populations exhibit specificity depending on the needs of the facilities. Interestingly, the ADFW viruses were associated with the anaerobic environments of engineered systems in the IMG/VR database, indicating the presence of anaerobic-specific viruses in ADFW systems and their possible association with anaerobic processes. Nevertheless, the emergence of novel viruses has brought attention to the issue of safety. AD residues are commonly returned to the land as a fertilizer [60]. Only conventional pathogenic bacteria are regulated in some environmental policies and standards [61]. However, the safety of extended viruses deserves further consideration.

Frequent fluctuations in viral and prokaryotic diversity were observed throughout the cultivation period in the lab-scale reactors, which indicated a constant evolution in microbial composition in response to changes in the external environment (Table S10). The viral community followed a similar evolutionary pattern to the prokaryotic community (Fig. 3e, f). Previous studies have revealed the adaptive strategies of functional microorganisms under ammonium [62] and LCFA stress [63, 64]. It is generally accepted that the external environment drives the evolution of prokaryotes. However, virus-mediated effects on prokaryotic community dynamics may have been overlooked.

The CRISPR-Cas spacer matching results suggested that viruses could infect a majority of phyla (Figure S6), whose functions involved hydrolytic acidification and methanogenesis in ADFW systems. The virus infection range may be narrow or extensive, and there is even the possibility of cross-domain infection, which widely occurs in other ecosystems [65]. The fact that only a fraction of the bacterial and archaeal genomes encodes the CRISPR system [66] makes it difficult to match all reliable hosts. However, the CRISPR-based approach to identifying hosts remains genetically reliable, although future validation is needed in combination with other host prediction methods, such as culture experiments or Hi-C metagenomics [65]. Overall, this study demonstrated to some extent the virus-host interactions in ADFW systems and attempted to interpret the infection modes of a number of functional microorganisms involved in methanogenesis.

The current life strategies of viruses include the “kill-the-winner” [67] and “piggyback-the-winner” models [68]. Viruses can regulate the dominant population in a community through predation or they inhibit lysis to maintain high host densities [67, 68]. Both of these patterns may be related to environmental factors and virus types [69]. In this study, the investigation into the infection patterns for SRB, SAOB, and methanogens under different environmental conditions suggested that hosts were often infected by multiple viruses and that the variation in abundances of the hosts and their infesting viruses can vary (Figure S7). Existing viral infestation models do not fully explain abundant species systems. It may be helpful to regulate the bioconcentration of ADFW systems through environmental factors, in order to explore viral infestation of specific species. In addition, highly resistant prokaryotes have a more stable intracellular environment under adverse conditions, which provides more suitable conditions for viruses to survive and reproduce [70]. This was reflected in SAOB and *Methanoculleus*, which both show positive effects in typical inhibition scenarios of ADFW systems [49, 50]. This trend provided a basis for future research on enhancing the adaptability of microorganisms related to the SAO-HM pathway through viral action in adverse environments.

Viruses can affect important metabolic functions in their hosts by carrying AMGs [71]. Biogas production and digestate nutrient availability are closely related to the conversion of carbon, nitrogen, and sulfur in AD systems. This study identified viruses that encoded enzymes for the degradation of LCFAs to key intermediates in LCFA-rich ADFW systems. Virus-mediated sulfur auxiliary metabolism widely exists in various ecosystems and its contributes to the global biogeochemical cycle [16]. The ASR and DSR are considered to be the main sulfate-reducing pathways and may be relate to the carbon cycle driven by SRB [72]. The involvement of viruses in the ASR and DSR pathway was first discovery in ADFW systems. It revealed the possibility that viruses participate in elemental cycles. Interestingly, the results from this study showed that viruses encoded part of the enzymes involved in the methanogenesis pathway, including the AM pathway and the SAO-HM pathway (Fig. 6). Hence, the importance of viruses in ADFW systems may have been underestimated. The goal of ADFW systems has gradually focused on the production of clean energy from the treatment of organic waste. Continuous and efficient operation has become the direction in which industrial ADFW systems continue to develop. Phage therapy has a wide range of applications in industry [73], agriculture [74], and medicine [75]. Artificial modification of the phage genome based on a comprehensive analysis of the viral properties of ADFW systems, combined with engineered phage design techniques [76], offers the potential for phage therapy to be applied to engineering technology associated with ADFW.

## Conclusions

The complex microbially based ecological patterns in ADFW systems meant that the effect of viruses on microbial population and ecology has not been thoroughly explored. In this study, in-depth mining of metagenomic data successfully revealed the existence of novel and diverse viral populations in practical engineering scenarios. The results have greatly expanded the potential diversity of viral populations in ADFW systems. While nearly half of viruses appeared to be unknown taxa, the gene-share networks suggested that there were anaerobic-specific ADFW viruses. The ADFW systems were shown to be the microbial pool whose community structure may be influenced by feed characteristics and other physicochemical parameters. Evidence from the dynamic analysis of multivariate scenarios at the lab scale showed that viruses and prokaryotes had similar kinetic characteristics. Many of the putative hosts belonged to key functional microorganisms, whose ecological niches can be regulated through viral infection behavior. In addition, virus-encoded AMGs, including carbon, sulfur, and fatty acid metabolism, may assist the metabolism of substances and even methanogenesis in the prokaryotic hosts during the infection period. In conclusion, our results highlighted the role of viruses played in the ecology of ADFW systems and provide support for improving material transformation efficiency in engineering applications.

### Supplementary Information

**Additional file 1.** 

**Additional file 2.** 

## Abbreviations

ADFW: Anaerobic digestion of food waste
AMGs: Auxiliary metabolic genes
sCOD: Soluble chemical oxygen demand
TOC: Total organic carbon
TN: Total nitrogen
TAN: Total ammonia nitrogen
VFAs: Volatile fatty acids
MAGs: Metagenome-assembled genomes
ORFs: Open reading frames
vOTUs: Viral operational taxonomic units
LCA: Lowest common ancestor
VCs: Viral clusters
TPM: Transcripts per kilobase of exon model per million mapped reads
PCoA: Principal coordinate analysis
MRM: Multiple regression matrix
OLR: Organic loading rate
MM: Methylotrophic methanogenesis
LCFAs: Long-chain fatty acids
WL: Wood-Ljungdahl
ASR: Assimilatory sulfate reduction
DSR: Dissimilatory sulfate reduction
SRB: Sulfate-reducing bacteria
SAOB: Syntrophic acetate-oxidizing bacteria
AM: Acetoclastic methanogenesis
SAO-HM: Syntrophic acetate oxidation coupled with hydrogenotrophic methanogenesis
