# Supplementary material for: Presence and role of viruses in anaerobic digestion of food waste under environmental variability
Source: Microbiome. 2023 Aug 4;11:170. doi: 10.1186/s40168-023-01585-z (PMC10401857; doi:10.1186/s40168-023-01585-z)
Supplement: Supplementary file 2 — Additional file 1. [file 40168_2023_1585_MOESM1_ESM.docx]

**Supporting Information**

**Presence and role of viruses in anaerobic digestion of food waste under environmental variability**

**Authors:** Lu Fan^a^, Wei Peng^a,b,c,*^, Haowen Duan^a^, Fan Lü^a,b,c^, Hua Zhang^a,b,c^, Pinjing He ^a,b,c,*^

**Affiliation:**

a Institute of Waste Treatment and Reclamation, Tongji University, Shanghai, 200092, China

b Shanghai Institute of Pollution Control and Ecological Security, Shanghai, 200092, China

c Shanghai Engineering Research Center of Multi-Source Solid Wastes Co-processing and Energy Utilization, Shanghai, 200092, China

***Correspondence to:** Pinjing He, email: solidwaste@tongji.edu.cn; Wei Peng, email: weipeng@tongji.edu.cn.

# **Supplementary Methods**

**Description S1. Operation and sampling of the full-scale ADFW reactors**

**Operation.** Received food waste (FW) in the studied FW treatment plant is divided into liquid-like FW and solid-like FW in the receiving hopper and conveyor belt, with liquid-like FW going directly into the pulping process. Solid-like FW is sorted by mechanical sorting, and then pulped. The crushed slurry is fed into the oil extraction unit, where is heated (65℃) and separated from the oil-water mixture by a three-phase separator. The separated oil-water mixture is again subjected to the thermal treatment process (85-90℃) and centrifuged to separate the oil. The liquid-like organic slurry from the two centrifugations is transferred to the anaerobic digestion (AD) reactors. The AD process applies five mesophilic continuously stirred tank reactors, operated at 35℃ with an effective volume of 600 m^3^ in a single reactor. The hydraulic retention time (HRT) of 35 days and design organic loading rate (OLR) of 2.5 kg-VS/m^3^·d (see **Figure SM1** for the flow of anaerobic digestion of food waste (ADFW) plant).

**Sampling.** Five full-scale reactor samples were taken from the sampling port at 18 m in each reactor. The sampling gate was opened and the 2-L sample was collected in a polyethylene bottle after the effluent has stabilized and homogenized. The samples were used partly for physicochemical and microbiological analysis and partly for inoculum of lab-scale reactors. The original feedstock of lab-scale reactors was taken from the liquid-like organic slurry before it is transported to the AD reactors.


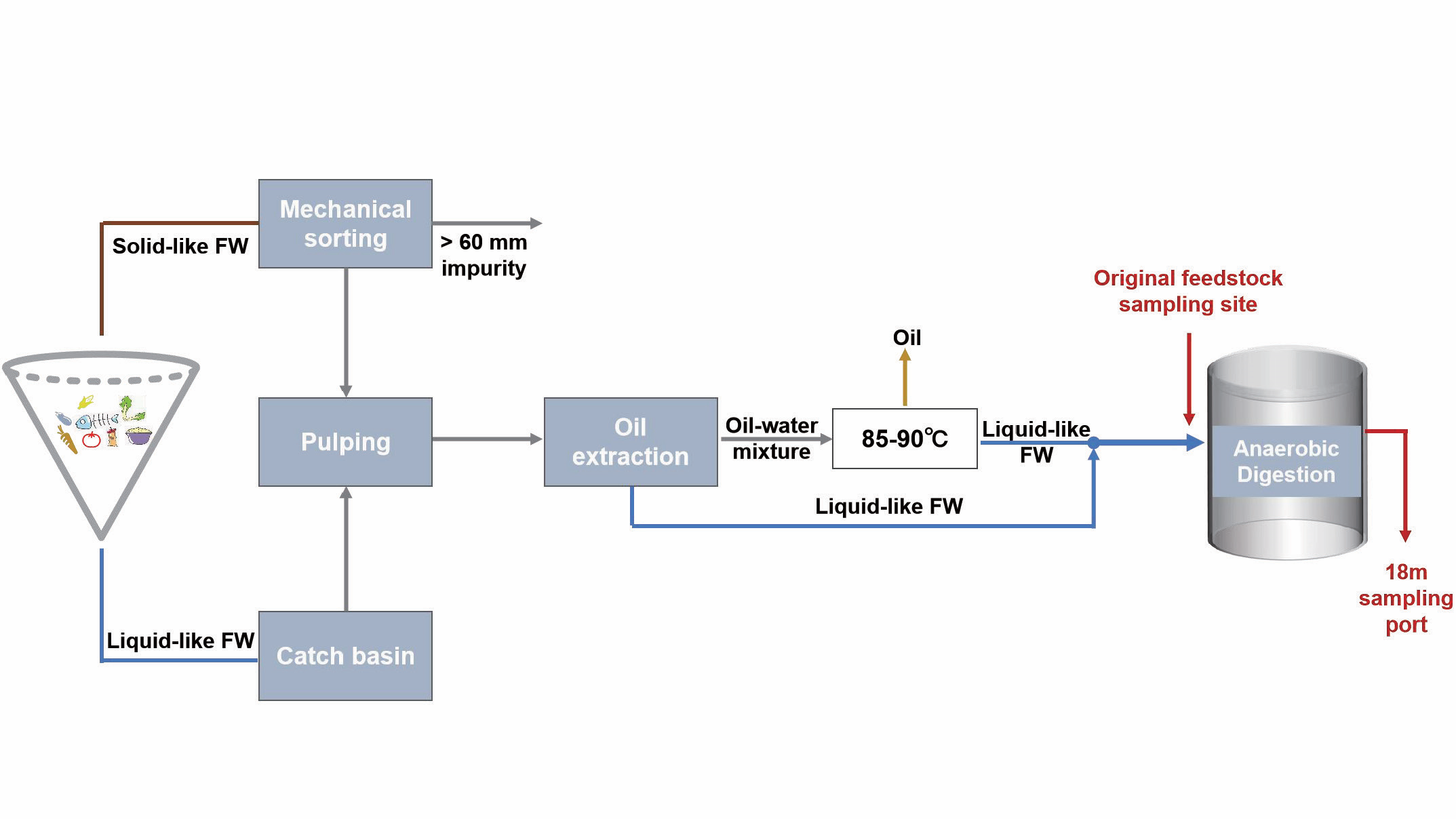


**Figure SM1.** Process flow of anaerobic digestion plant for food waste.

**Description S2. Lab-scale ADFW reactors incubation**

**Start-up.** Lab-scale incubation started in 2-L (actual working volume was 1.8 L) continuously stirred tank reactors, which sealed by a rubber plug and equipped with feed ports, discharge ports, and a continuous stirring device. Five full-scale reactor samples were mixed and injected into three lab-scale reactors at 1.8 L. The reactor headspace was then filled with N_2_ (99.9999%) to remove the internal air. Gas bag was connected to receive the gas production. After the system was built, it was incubated in a thermostat at 38.0 ± 0.5 °C (BPC Instruments AB, Lund, Sweden) (see **Figure SM2** for the schematic diagram of the incubation device).

**Incubation.** After the start-up of the reactor on September 27, 2021, a total of 12 days of starvation incubation was carried out until the organic matter in the inoculum was consumed. From October 9, 2021 (labelled the 0^th^ day), the three reactors were fed and incubated for 126 days in four stages. Stage I lasted for 44 days, with three reactors fed with original feedstock to maintain stable operation. The initial feedstock volume was 17 mL, resulting in an OLR of 0.5 g-VS/L·d. Through observation of the biogas production effect, a moderate increase in organic load was beneficial to the efficient operation of the reactor by increasing the feedstock volume. The OLR was ultimately raised to 0.83 g-VS/L·d. The OLR was kept at 0.83 g-VS/L·d in Stage II until 92nd day. C-group has always been fed on original feedstock. For N-group, NH_4_Cl was added to the feedstock to increase the concentration of ammonium in the reactor. Palmitate was the most abundant LCFA salts in the lipid-rich waste, which has been used as a typical lipid source in studies on AD [1]. Based on the study of Sousa et al. [2], the Methanospirillum hungatei IC50 was 1 mmol/L for palmitate. Considering that the mixed system of ADFW is different from pure culture conditions, and part of unhydrolyzed lipids are separated in the oil extraction unit. In addition, to ensure the continuity of the experiment, a gradient lifting was used to increase the concentration of LCFA. Hence, 0.1 mmol/L palmitate was added as the lipid supply to the feedstock of L-group in Stage II. The feedstock volume of Stage III and Stage IV was 40 and 50 mL, allowing OLR was operated at 1.04 and 1.3 g-VS/L·d, respectively. N-group was moderately control of NH_4_Cl addition to maintain the concentration of TAN at 6000 to 8000 mg/L, which is a potentially high ammonium condition in full-scale reactors and has the ability to maintain microbial dynamics. For L-group, the concentration of added palmitate was increased twice to 0.4 mmol/L. The specific daily feeding information shown in **Table S1**.

**Sampling and feeding.** Biogas was collected in the sealed gas sampling bags. The sampling bags was emptied after each volume measurement. A sterile injection syringe connected to a rubber catheter was used to collect solid-liquid mixture through the outlet. After sampling, the feedstock was replenished through the reactor inlet. The sampling volume is the same as the feed volume, ensuring that the working volume in the reactor is maintained at 1.8 L. The interval period was adjusted timely according the trend in gas production. Details about the specific dates shown in **Table S5**.


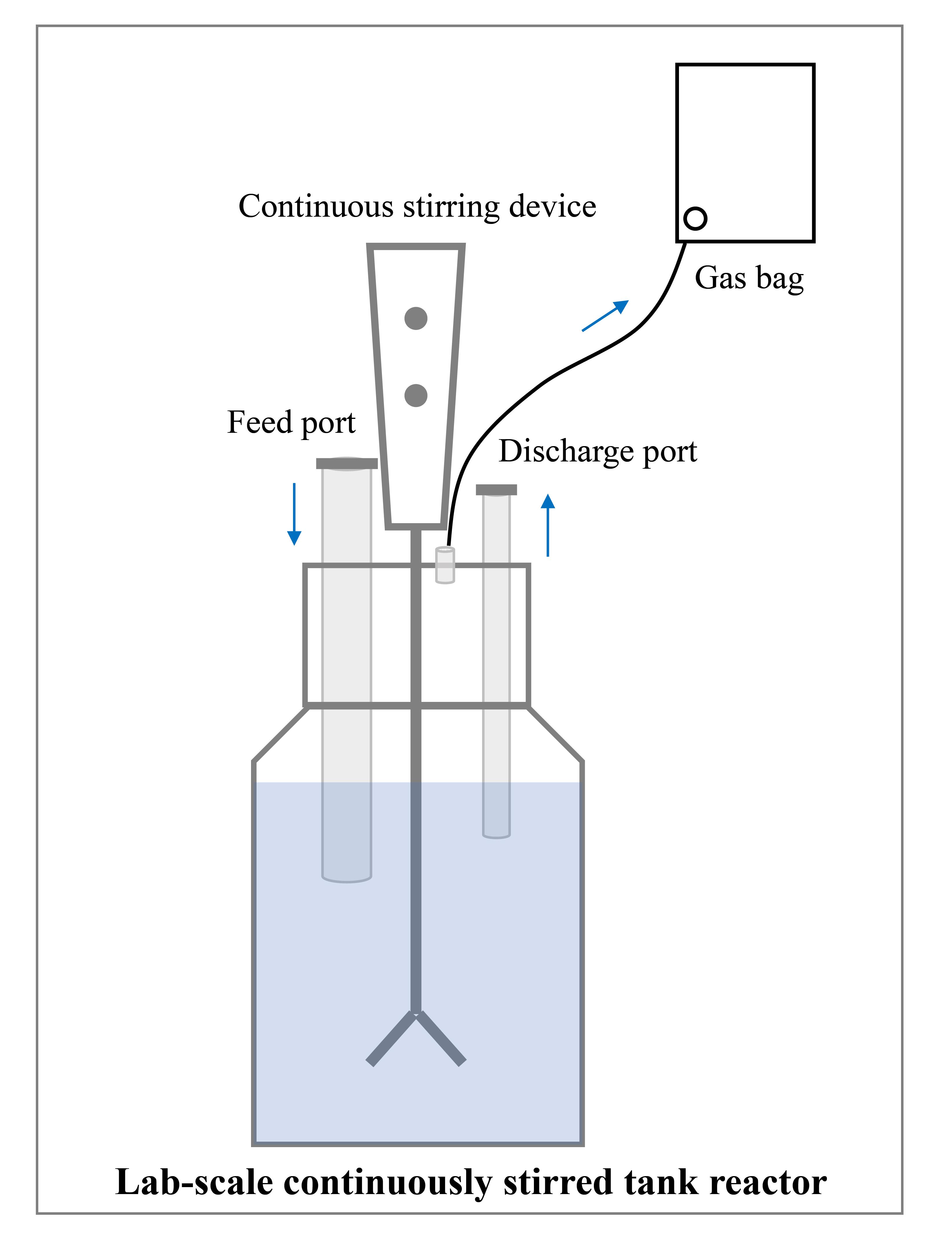


**Figure SM2.** Schematic of lab-scale anaerobic digestion reactor device.

**Description S3.** **Physiochemical properties measurements**

Biogas volume was measured using the peristaltic pump with a gas meter. Biogas components (CH_4_ and CO_2_) were measured using biogas analyzer (Geotech, Britain) which connected to reactor for real-time measurement during sampling. Fresh samples were immediately transferred out of the section for pH determination by using a pH meter (PHS-3BW, Bante Instruments). Samples were centrifuged at 15,000 rpm for 10 min, and the supernatant was obtained for the analysis of soluble chemical oxygen demand (sCOD), total organic carbon (TOC), total nitrogen (TN), total ammonia nitrogen (TAN) and volatile fatty acids (VFAs). The supernatant was added to the HACH COD reagent kit (20-1500 mg/L COD, HACH, USA) for sCOD measurement. The digestion was carried out at 150 ℃ for 2 hours using the digestion instrument (DRB200, HACH, USA), and then a spectrophotometer (DR3900, HACH, USA) was used to determined sCOD by colorimetry. Similarly, the TAN of supernatant was measured using HACH nitrogen-ammonia reagent set with salicylate method (0.01-0.50 mg/L NH_3_-N, HACH, USA). According to the operating manual, samples were added to the reagent tubes followed by separate reagent packs of ammonia salicylate and ammonia cyanurate. Mix thoroughly and leave to react for 20 min, the results were measured using a spectrophotometer (DR3900, HACH, USA). TOC and TN were measured in a total organic carbon analyzer (TOC-V CPH, Shimadzu, Japan) which adopting 680 ℃ combustion catalytic oxidation method and matching TN unit (TNM-L). The supernatant was diluted and acidified to pH of 2 for VFAs analysis. The concentrations of carboxylates (C2-C7) were measured by a gas chromatography equipped with a flame ionization detector (Trace 1300, Thermo Fisher Scientific, Waltham, USA), and a DB-WAX UI column with 30 m length × 0.25 mm I.D. × 0.25 μm film thickness (Agilent, Santa Clara, USA) was used to separate component.

**Description S4. DNA extraction and metagenomic sequencing**

**DNA extraction.** Samples of each group were collected for DNA extraction after 0, 44, 92, 108 and 126 days of incubation (labelled the 0th, 44th, 92nd, 108th and 126th days, respectively). 2-mL of each sample was collected in a tube and centrifuged at 15,000 rpm and 4 ℃ for 15 min. After centrifugation, the supernatant was removed and the sediment at the bottom of the centrifuge tube was used for DNA extraction using PowerSoil^TM^ DNA isolation kit (MoBio Laboratories Inc, CA). The Solution, PowerBead Pro Tubes, MB Spin Column, Collection Tube and Elution Tube used in the extraction process were obtained from the PowerSoil^TM^ DNA isolation kit (MoBio Laboratories Inc, CA).

1. Add 800 μl Solution CD1 to the centrifuge tube with sediment and cortex briefly to mix. Transfer of the mixture to the PowerBead Pro Tubes which have beads in the tubes.
2. Secure the PowerBead Pro Tubes horizontally on a vortex adapter (Precellys 24, Bertin, France). Vortex three times at maximum speed for 60s at 5 min intervals and leave to cool in a refrigerator at 4°C during the interval.
3. Centrifuge the PowerBead Pro Tubes at 15,000 g for 1 min and transfer the supernatant to the clean 2 ml centrifuge tube.
4. Add 200 μl Solution CD2 and vortex for 5s.
5. Centrifuge the centrifuge tube at 15,000 g for 1 min. Transfer up to 700 μl of supernatant to the clean 2 ml centrifuge tube.
6. Add 600 μl Solution CD2 and vortex for 5s.
7. Load 650 μl of the lysate onto an MB Spin Column and centrifuge at 15,000 g for 1 min.
8. Discard the flow-through and repeat step 7 to ensure that all of the lysate has passed through the MB Spin Column. And carefully place the MB Spin Column into a clean 2 ml Collection Tube.
9. Add 500 μl of Solution EA to the MB Spin Column. Centrifuge at 15,000 g for 1 min. And discard the flow-through and place the MB Spin Column back into the same 2 ml Collection Tube.
10. Add 500 μl of Solution C5 to the MB Spin Column. Centrifuge at 15,000 g for 1 min. Discard the flow-through and place the MB Spin Column into a new 2 ml Collection Tube
11. Centrifuge at up to 16,000 g for 2 min. And discard the flow-through and place the MB Spin Column back into a new 1.5 ml Elution Tube.
12. Add 50-100 μl of Solution C6 to the center of the white filter membrane.
13. Centrifuge at 15,000 g for 1 min. Discard the MB Spin Column. The DNA is collected in the 1.5 ml Elution Tube. The extracted DNA was stored at -40 ℃.

**Metagenome Sequencing.** A total amount of 0.2 μg DNA per sample was used for DNA library preparations. Sequencing library was generated using NEBNext® UltraTM DNA Library Prep Kit for Illumina (New England Biolabs, USA) following manual and index codes were added to each sample. Briefly, genomic DNA was fragmented by sonication to a size of 350 bp. Then DNA fragments were end-polished, A-tailed, and ligated with the full-length adapter for Illumina sequencing, followed by further PCR amplification. After PCR products were purified by AMPure XP system (Beverly, USA). Subsequently, library quality was assessed on the Agilent 5400 system (Agilent, USA）and quantified by qPCR (1.5 nM). The qualified libraries were pooled and sequenced on Illumina platforms with PE150 strategy in Novogene Bioinformatics Technology Co., Ltd (Beijing, China).

**Description S5. Metagenomic quality control and assembly**

**Quality Control.** The quality control of the raw reads was performed using Fastq (v 0.19.7) [3]. Paired-end reads that contain adapter contamination were discarded. And the reads which have more than 10% of bases are uncertain or over 50% of bases are low quality (Phred quality < 5) in either one read were filtered. Finally, the Read_QC module (parameters: --skip-bmtagger) within metaWRAP (v1.3.2) [4] was used for further trimming. The paired-end reads were trimmed according to the Phred score with default setting of Trim-galore, ensuring that only high-quality sequences were retained. Human genome alignment was chosen to be skipped as no human contaminated reads were considered to be present in this study.

**Assembly.** After quality control, the ASSEMBLY module within metaWRAP (v1.3.2) [4] was used to assemble the clean reads. The assembly method was performed using metaSPAdes v3.10.0 (kmer size: 21, 33, and 55) (parameter: -m 200; set other parameters as default). Total 20 single samples, include 5 full-scale reactor samples and 15 lab-scale reactor samples, were individually assembled. The assembled contigs of each sample longer than 1000 bp were used for further analyses.

**Description S6. Metagenomic binning and taxonomy and function annotation of prokaryote**

**Binning.** For assembly binning, contigs were binned using the Binning module (parameter: --metabat2 --maxbin2 --concoct --run checkm; set other parameters as default) within metaWRAP (v1.3.2) [4] to produce three bin sets for each assembly. The Bin_refinement module (parameter: -c 50 -x 10; set other parameters as default) within metaWRAP (v1.3.2) [4] was used to create hybridized bins and evaluate the quality of the bins in each of the original and hybridized bin sets, resulting in a consolidated and improved bin set of each sample. All the bins of fifteen lab-scale samples were dereplicated to produce lab-scale AD prokaryotic metagenome assembled genomes (MAGs) dataset using dRep (v3.2.2) (parameter: -comp 50 -con 10 -sa 0.95; set other parameters as default) [5].

**Taxonomy and function annotation of the MAGs.** The dereplicated MAGs were annotated based on the Genome Taxonomy Database (GTDB) taxonomy (release 05-RS95) using GTDB-Tk (v1.7.0) (classify_wf module) [6]. The taxonomic classifications of the MAGs were based on GTDB taxonomy. Phylogenetic tree of the MAGs constructed by GTDB-TK was visualized using iTOL (v6.5.8) [7]. Genes and proteins sequences of each MAGs were predicated by Prodigal (v2.6.3) (parameter: -p meta; set other parameters as default) [8], and the functions of theses gene were annotated using EnrichM (v0.6.4) (annotate module; --ko; set other parameters as default) (https://github.com/geronimp/enrichM). The Kyoto Encyclopedia of Genes and Genome (KEGG) orthologous group ids (KOs) of genes were used for metabolic reconstruction. The functions of the MAGs associated with methanogenesis were analyzed manually based on taxonomic assignment and metabolic pathways.

**Supplementary Figures**





**Figure S1.** Taxonomic assignments of vOTUs in five full-scale reactors. The label “Unclassified Family” means the vOTU could not be attributed to a specific family, but can be annotated at genus or species level. The label “Others” represented the total relative abundance of remaining taxa.


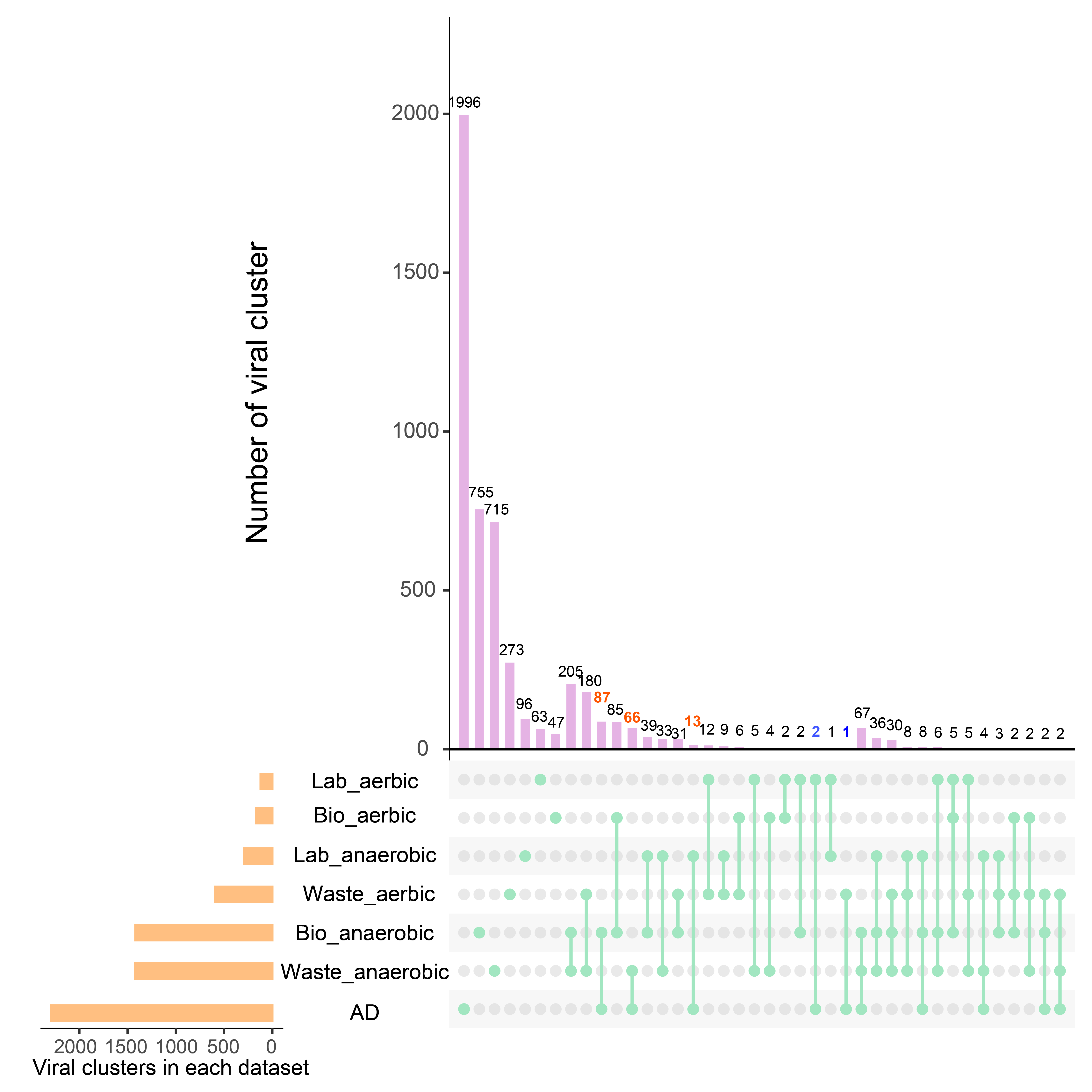


**Figure S2.** The UpSet chart of shared viral clusters among ADFW and other ecosystems. The numbers of shared clusters between ADFW and anaerobic environments, ADFW and aerobic environments are labeled orange and blue, respectively. “Lab”, “Bio”, and “Waste” denoted the IMG/VR v3.0 taxonomy units: lab enrichment, bioreactor, and wastewater.


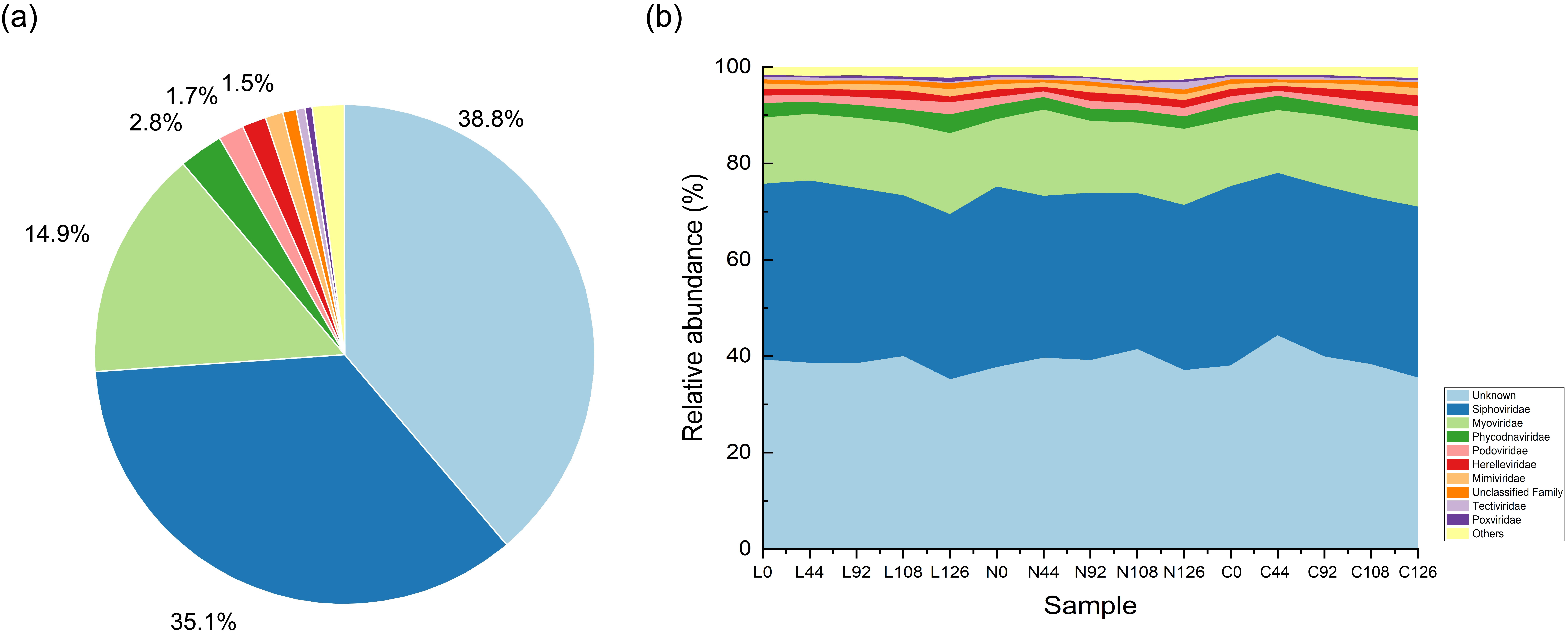


**Figure S3.** Relative abundance (%) of viral taxa at family level with predicted annotation in lab-scale reactors.


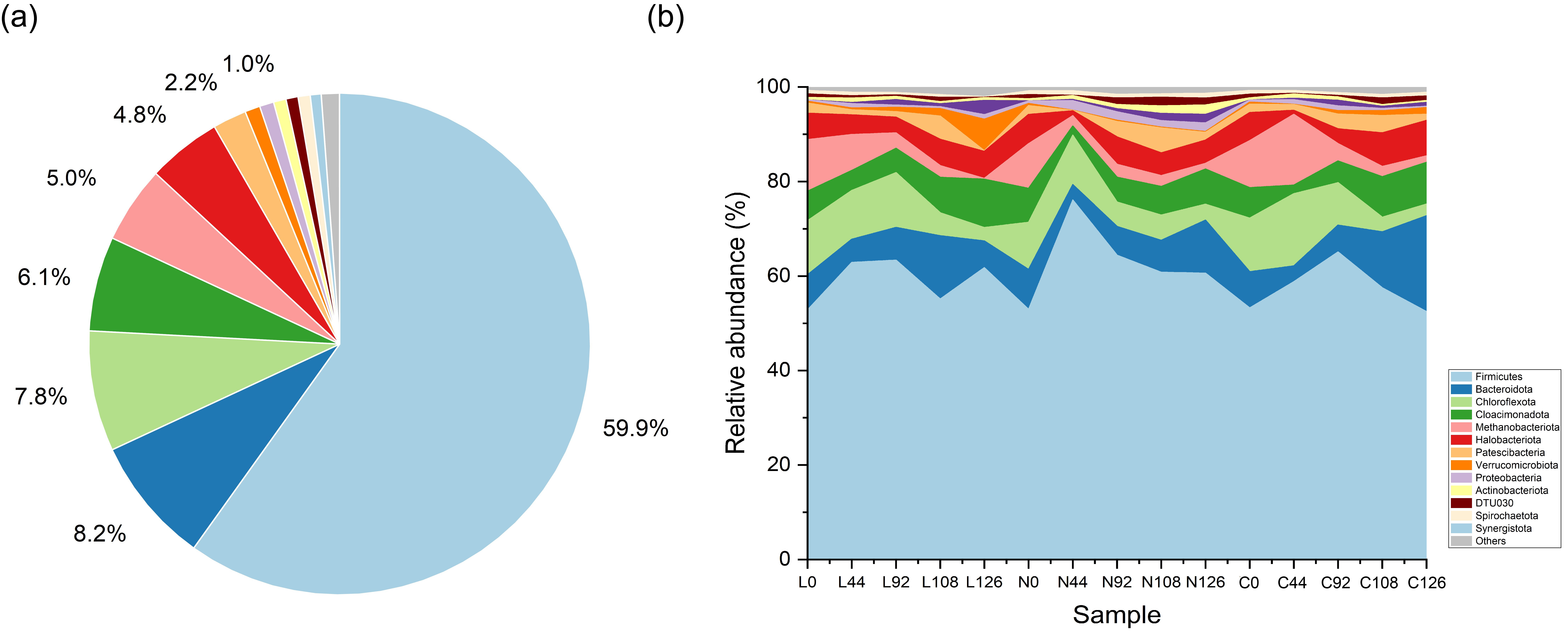


**Figure S4.** The charts show the relative abundance (%) of prokaryotes taxa at phylum level in lab-scale reactors. “Others” denotes the total relative abundance of remaining taxa.


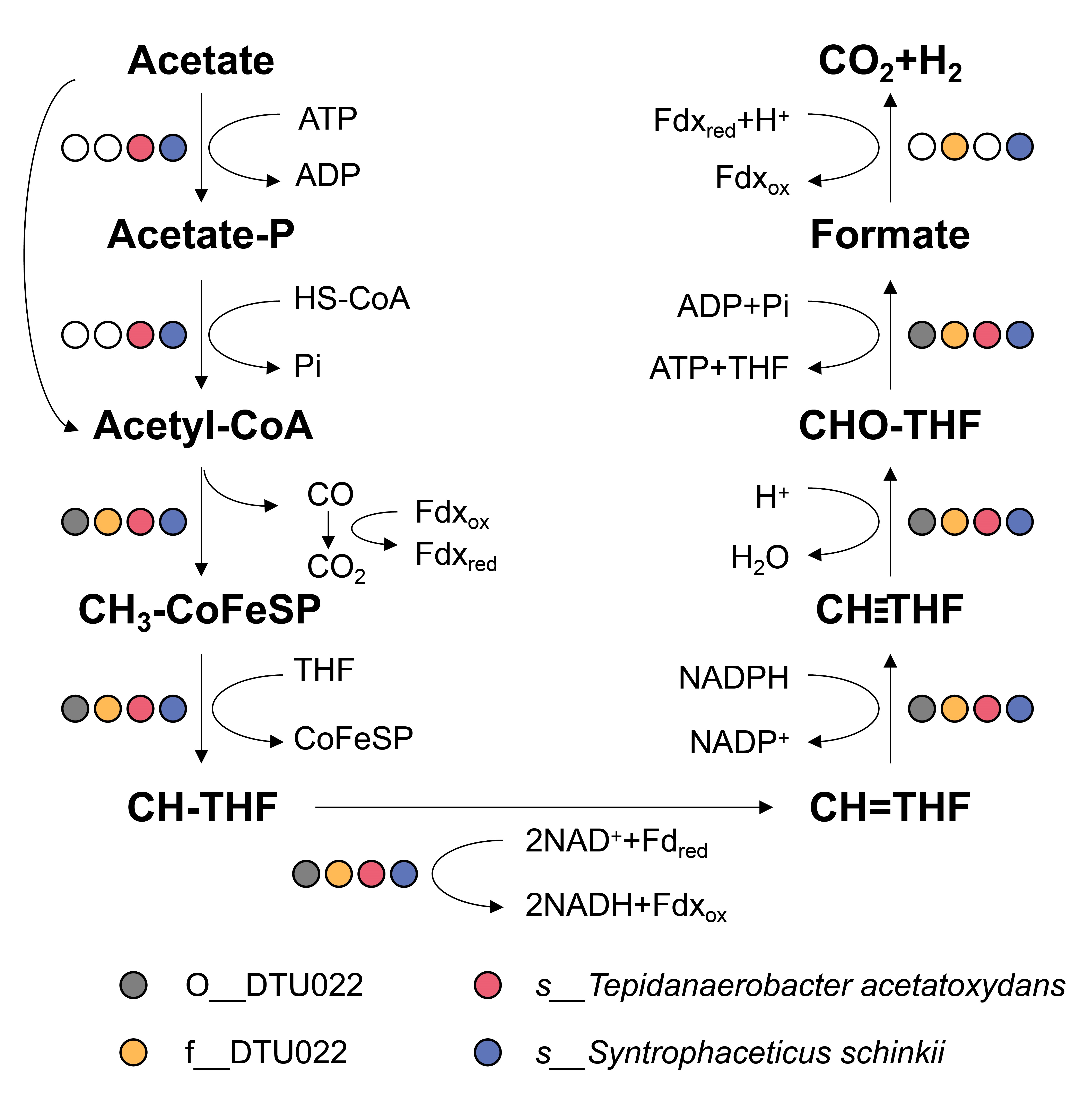


**Figure S5.** The metabolic pathway of acetate oxidation through the oxidative Wood-Ljungdahl (WL) pathway.


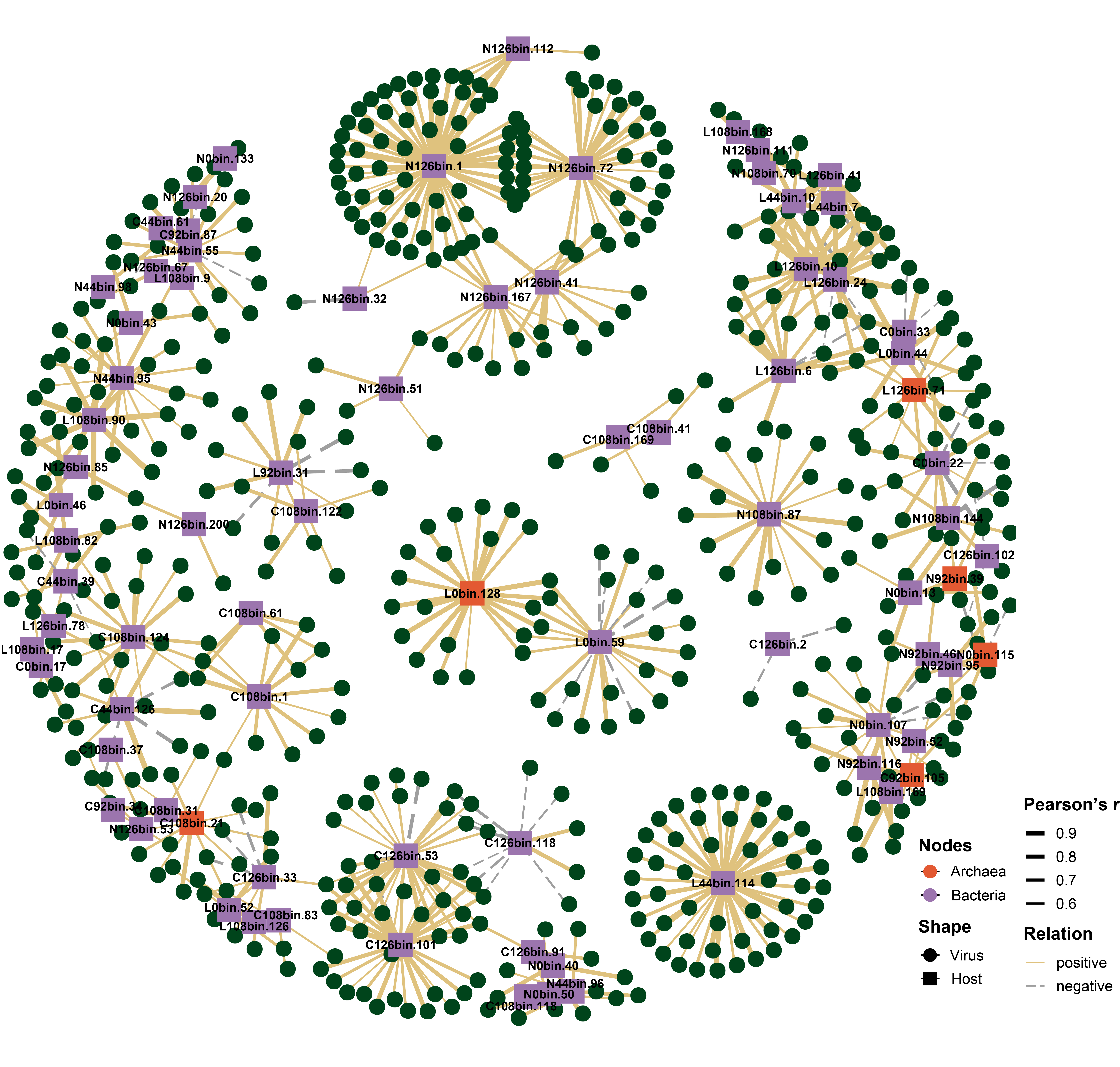


**Figure S6.** A network representing CRISPR-based interactions from all samples involving the viruses (circle nodes) and their hosts (square nodes). The edges represent the Pearson correlation between viruses and their hosts.





**Figure S7.** Networks of key functional microorganisms and viruses interactions. (a) SRB; (b) SAOB; (c) Archaea. The circle nodes represent viruses and square node are used to indicate hosts. The edges linking two points are calculated by Pearson correlation.





**Figure S8.** Pearson correlations between SAOB and their viruses across 15 samples.

**Supplementary Tables**

**Table S1:** Details of feed information for semi-continuous lab-scale reactors.

**Table S2:** Details information of vOTUs identified in five full-scale reactors.

**Table S3:** Relative abundance and taxonomy affiliation of vOTUs identified in five full-scale reactors.

**Table S4:** Viral clusters between viral sequences of full-scale reactors and NCBI Refseq viral genomes.

**Table S5:** Physicochemical parameters during cultivation in three lab-scale reactors.

**Table S6:** Details information of vOTUs identified in lab-scale reactors.

**Table S7:** Relative abundance and taxonomy affiliation of vOTUs identified in lab-scale reactors.

**Table S8:** Relative abundance and taxonomy affiliation of MAGs in lab-scale reactors.

**Table S9:** Pearson correlation between viral taxa and physicochemical parameters.

**Table S10:** Diversity index of viral and prokaryotic communities.

**Table S11:** Mantel test of biotic and physicochemical parameters.

**Table S12:** The influence factors on prokaryotic community composition calculated by MRM.

**Table S13:** Metabolic information of identified SAOB performing WL pathway.

**Table S14:** Pearson correlation between viruses and their hosts.

**Table S15:** Characteristics of viruses infected functional microorganisms related to methanogenesis.

**Table S16:** Metabolism information of identified vOTUs in lab-scale.

**Table S17:** Details about selected vOTUs performing methanogenic pathway.

**References**

1. Cirne DG, Paloumet X, Björnsson L, Alves MM, Mattiasson B. Anaerobic digestion of lipid-rich waste—Effects of lipid concentration. Renew Energy. 2007;32:965–75.

2. Z SD, F SA, Juliana R, P GA, Sï¿½nia B, M SAJ, et al. Activity and Viability of Methanogens in Anaerobic Digestion of Unsaturated and Saturated Long-Chain Fatty Acids. Appl Environ Microbiol. 2013;79:4239–45.

3. Chen S, Zhou Y, Chen Y, Gu J. fastp: an ultra-fast all-in-one FASTQ preprocessor. Bioinformatics. 2018;34:i884–90.

4. Uritskiy G V, DiRuggiero J, Taylor J. MetaWRAP—a flexible pipeline for genome-resolved metagenomic data analysis. Microbiome. 2018;6:158.

5. Olm MR, Brown CT, Brooks B, Banfield JF. dRep: a tool for fast and accurate genomic comparisons that enables improved genome recovery from metagenomes through de-replication. ISME J. 2017;11:2864–8.

6. Chaumeil P-A, Mussig AJ, Hugenholtz P, Parks DH. GTDB-Tk: a toolkit to classify genomes with the genome taxonomy database. Bioinformatics. 2019;36:1925–7.

7. Letunic I, Bork P. Interactive Tree Of Life (iTOL) v5: an online tool for phylogenetic tree display and annotation. Nucleic Acids Res. 2021;49:W293–6.

8. Hyatt D, Chen G-L, LoCascio PF, Land ML, Larimer FW, Hauser LJ. Prodigal: prokaryotic gene recognition and translation initiation site identification. BMC Bioinformatics. 2010;11:119.
